# VAV2 orchestrates the interplay between regenerative proliferation and ribogenesis in both keratinocytes and oral squamous cell carcinoma

**DOI:** 10.1038/s41598-024-54808-0

**Published:** 2024-02-19

**Authors:** Natalia Fernández-Parejo, L. Francisco Lorenzo-Martín, Juana M. García-Pedrero, Juan P. Rodrigo, Mercedes Dosil, Xosé R. Bustelo

**Affiliations:** 1 Centro de Investigación del Cáncer and Instituto de Biología Molecular del Cáncer, CSIC and Universidad de Salamanca, 37007 Salamanca, Spain; 2 Centro de Investigación Biomédica en Red de Cáncer, 28029 Madrid, Spain; 3 Hospital Universitario Central de Asturias and Instituto de Investigación Sanitaria del Principado de Asturias (ISPA), Instituto Universitario de Oncología del Principado de Asturias, University of Oviedo, 33011 Oviedo, Spain; 4 Present Address: Laboratory of Stem Cell Bioengineering, École Polytechnique Fédérale de Lausanne, 1015 Lausanne, Switzerland

**Keywords:** Guanosine nucleotide exchange factors, RHO GTPases, RAC1, RHOA, PAK1, ROCK, YAP/TAZ, Ribosomes, RNA polymerase I, Patient-derived cells, Patient prognosis, Oncogenic signaling, Squamous cell carcinoma, Head and neck cancer, Oncogenes, Oral cancer, RHO signalling, GTP-binding protein regulators, Phosphoproteins, Ribosomal proteins

## Abstract

VAV2 is an activator of RHO GTPases that promotes and maintains regenerative proliferation-like states in normal keratinocytes and oral squamous cell carcinoma (OSCC) cells. Here, we demonstrate that VAV2 also regulates ribosome biogenesis in those cells, a program associated with poor prognosis of human papilloma virus-negative (HPV^−^) OSCC patients. Mechanistically, VAV2 regulates this process in a catalysis-dependent manner using a conserved pathway comprising the RAC1 and RHOA GTPases, the PAK and ROCK family kinases, and the c-MYC and YAP/TAZ transcription factors. This pathway directly promotes RNA polymerase I activity and synthesis of 47S pre-rRNA precursors. This process is further consolidated by the upregulation of ribosome biogenesis factors and the acquisition of the YAP/TAZ-dependent undifferentiated cell state. Finally, we show that RNA polymerase I is a therapeutic Achilles’ heel for both keratinocytes and OSCC patient-derived cells endowed with high VAV2 catalytic activity. Collectively, these findings highlight the therapeutic potential of modulating VAV2 and the ribosome biogenesis pathways in both preneoplastic and late progression stages of OSCC.

## Introduction

Head and neck squamous cell carcinoma (HNSCC) can develop in the epithelia of the mucosal lining of the upper aerodigestive tract areas, such as the oral epithelium, the tongue, the larynx, and the hypopharynx. These tumors are clinically challenging due to epidemiological incidence, metastatic properties, frequent posttreatment recurrence events, and limited therapeutic options. Factors influencing the development of these tumors include alcohol intake, tobacco smoking, and human papilloma virus (HPV) infections^1^. Approximately 670 000 HNSCC cases were detected worldwide in 2020, with an average mortality rate of 40–50%^2^.

Numerous biological traits favor HNSCC development and malignant properties^1,3^. One of them is regenerative proliferation, a feature characterized by the presence of high percentages of proliferating and undifferentiated cells that correlates with poor HNSCC prognosis^1,4^. This state can be orchestrated in a concerted manner by multiple transcription factors such as the YAP/TAZ complex, AP1, E2F, c-MYC, TP63, and ACTL6A^5–10^. Recently, the guanosine nucleotide exchange factor (GEF) VAV2 has been shown to have critical roles in early signaling events that trigger and maintain regenerative proliferation in normal keratinocytes and OSCC, respectively^11^. VAV2 is regulated by tyrosine phosphorylation and can catalyze the activation step of the GTPases RAC1 and RHOA^11–14^. Of note, VAV2 regulates this process by activating RAC1 and RHOA, the proximal GTPase effectors PAK and ROCK, and the transcription factors c-MYC and YAP/TAZ^11^. Those two transcription factors are eventually responsible for the high proliferative capacity (mediated by c-MYC) and the undifferentiated state (mediated by YAP/TAZ) shown by OSCC cells^11^. *VAV2* mRNA levels and VAV2-regulated gene signatures directly correlate with poor prognosis of HPV^−^ HNSCC patients, further underscoring the importance of this pathway for the malignant properties of this tumor type^11^. In line with this, xenotransplantation experiments have shown that knocking down endogenous *VAV2* reduces the primary tumorigenesis and metastatic properties of OSCC patient-derived cells (PDCs)^11^.

Recent evidence indicates that HNSCCs also depends on high ribosome biogenesis rates for optimal fitness^15^. This biological process is initiated by the RNA polymerase I-mediated transcription of the 47S pre-ribosome RNA (rRNA) in the nucleolus. This precursor subsequently undergoes several steps of cleavage and maturation in the nucleolus, nucleoplasm, and cytosol that eventually leads to the generation of the rRNAs that form part of either the small (18S rRNA) or large (28S rRNA, 5.8S rRNA) ribosome subunits. This maturation process is accompanied by the sequential docking and release of ribosome biogenesis factors and the final incorporation of ribosomal proteins^16,17^. This process is targeted by many oncogenic drivers and signal transduction pathways to promote the growth of cancer cells^16^. Connected to this route, the translation of specific transcript subsets via deregulation of translational regulators also contributes to HNSCC fitness^18–20^.

Despite this progress, we still have limited information regarding the potential interconnections established by all the foregoing biological pathways in HNSCC. For example, despite the extensive functional characterization of RHO proteins at the signaling and cellular level during the last decades, we know little about their influence on ribosome biogenesis in HNSCC and other tumor types. Recent observations indicate that such interconnections might indeed exist, although they appear to entail non-canonical and tumor type-specific signaling mechanisms. For example, in non-small cell lung cancer, the RHO GEF ECT2 and RAC1 promote direct RNA polymerase I transcription through interactions with the nucleolar protein nucleophosmin^21^. Conversely, a negative regulator of RHO GTPases (ARHGAP30) has been shown to negatively regulate ribogenesis in cervical cancer. This mechanism relies on the ubiquinylation-mediated degradation of a key ribosome biogenesis factor rather than on the expected regulation of RHO GTPase activity^22^. To date, however, we do not know whether these or other alternative mechanisms operate in HNSCC and its OSCC subtype. Potential interconnections between ribogenesis and regenerative proliferation also remain poorly characterized.

Using organotypic cultures of primary keratinocytes and OSCC PDCs as experimental model, we here present evidence demonstrating that VAV2 coordinates the concurrent regulation of regenerative proliferation and ribogenesis in a RAC1 and RHOA GTPase-dependent manner in those two cell types. This connection is therapeutically relevant, since the VAV2-regulated gene signature for ribosome biogenesis factors is associated with poor prognosis of HPV^−^ OSCC patients. Perhaps more importantly, we show that ribogenesis represents a key therapeutic vulnerability for keratinocytes and OSCC PDCs that have high levels of VAV2 activity.

## Results

### VAV2^Onc^-driven epidermal hyperplasia correlates with enhanced ribogenesis

We previously found using in silico annotation analyses that ribosome ribogenesis is one of the top upregulated biological functions in the hyperplasic epidermis of *Vav2*^Onc/Onc^ mice^11^. These knock-in mice endogenously express a truncated version of VAV2 (Δ1−186; herein, VAV2^Onc^) that shows constitutive catalytic activity due to the removal of the two autoinhibitory N-terminal domains. The expression of this mutant protein leads to the chronic stimulation of the downstream RAC1 and RHOA GTPases^11^. In line with those in silico analyses, we found using gene set enrichment analyses (GSEA) that gene signatures for ribosome biogenesis factors (Supplementary Fig. 1A and B) as well as for structural ribosomal proteins (Supplementary Fig. 1C and B) are highly enriched in the VAV2^Onc^-upregulated transcriptome. Further, the expression levels of the VAV2^Onc^-regulated transcripts for ribosome biogenesis factors were also found increased in OSCC as compared to either healthy or dysplastic tissue samples (Supplementary Fig. 1D, top left panel). The expression levels of the subset of VAV2^Onc^-regulated ribosome biogenesis factor-encoding transcripts also correlate with the abundance of the *VAV2* mRNA (Supplementary Fig. S1E, top middle panel) and with poor patient prognosis (Supplementary Fig. S1F, top right panel) when tested in a previously defined cohort of HPV^−^ OSCC patients^23^. The stratification power of this signature (*P* = 0.016) is similar to that provided by the expression of the *EGFR* transcript (*P* = 0.011), a gene with key protumorigenic functions in HNSCC^1,3^. However, its power is significantly lower than that provided by the expression levels of the *VAV2* mRNA itself (*P* = 0.008)^11^ as well as of other raw (*P* = 0.009) or refined (*P* < 1 × 10^−5^) VAV2^Onc^-regulated gene signatures^11^. Of note, the ribosome biogenesis gene signature is not effective at stratifying patients if the gene expression datasets lack information on HPV status (L.F.L.-M. and X.R.B., data not shown), suggesting that its functional relevance is limited to HPV^−^ HNSCC cases. Using these gene expression datasets, we observed no correlations between the gene signature levels for structural components of mature ribosomes and tumor progression (Supplementary Fig. 1D, bottom left panel), *VAV2* mRNA abundance (Supplementary Fig. 1D, bottom middle panel), or patient prognosis (Supplementary Fig. 1C, bottom right panel). Overall, these analyses suggest that ribogenesis upregulation might play pathogenic roles in VAV2^Onc^-dependent HPV^−^ OSCC subtypes.

**Figure 1:**
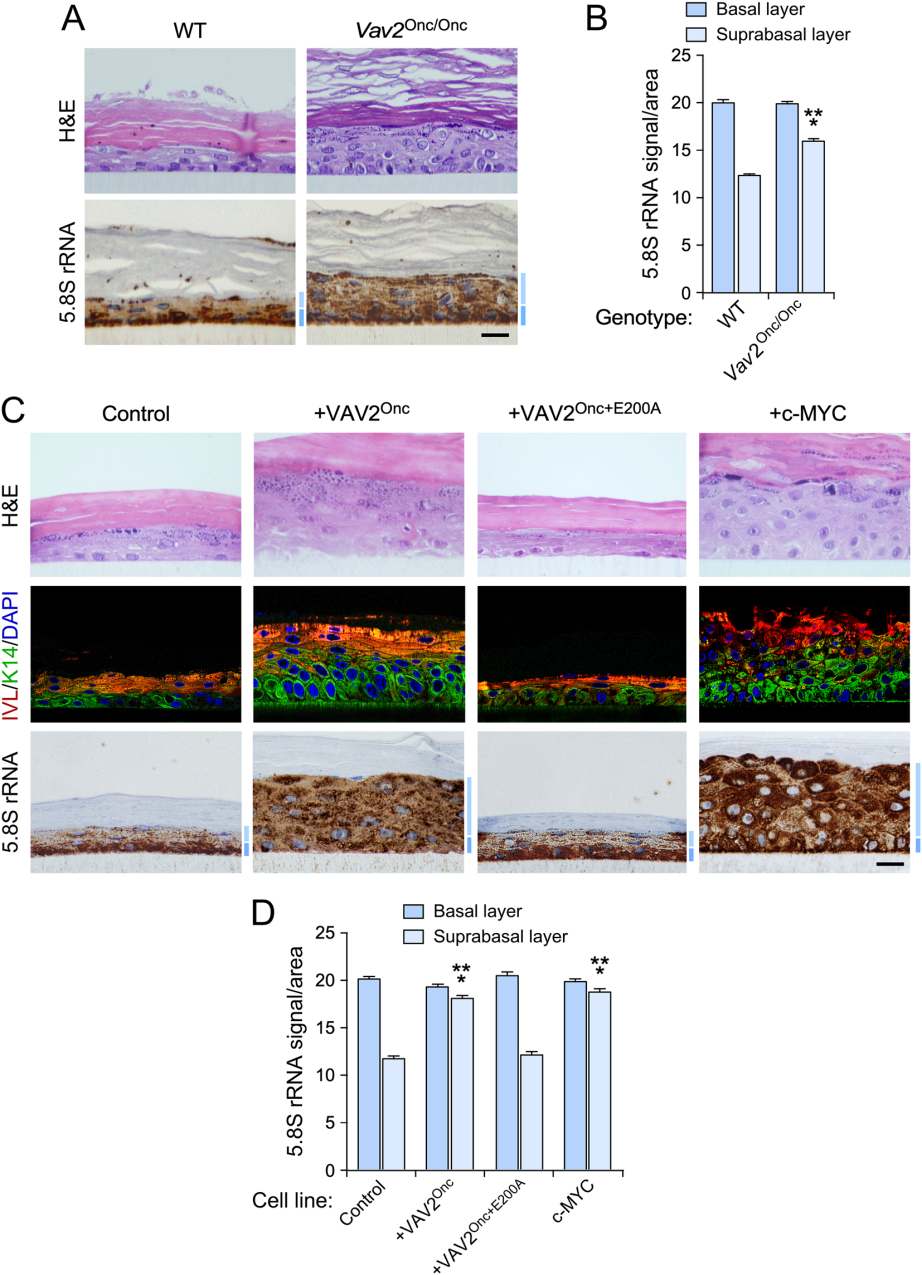
VAV2^Onc^-driven epidermal hyperplasia correlates with enhanced ribogenesis. (**A**) Histological sections from organotypic cultures using primary keratinocytes from wild-type (WT) and *Vav2*^Onc/Onc^ (*Vav2*^Onc/Onc^) mice that were stained with either H&E (top) or labeled with antibodies to the 5.8S rRNA antibody plus hematoxylin. The basal and suprabasal epidermal layers are indicated by a dark blue or a light blue bar (bottom panel), respectively. Scale bar, 10 μm. (**B**) Quantification of the 5.8S rRNA immunoreactivity obtained in the basal and suprabasal layers from (**A**). ***, *P* < 0.0001 (Student’s *t*-test, *n* = 3 independent cultures). (**C**) Tissue sections of 3D organotypic models using human keratinocytes expressing the indicated proteins upon staining with either H&E (top panels) or antibodies to involucrin (IVL, red color), keratin 14 (K14; green, middle panels), and the 5.8S rRNA (brown, bottom panels). Some sections were subsequently counterstained with either DAPI (blue; middle panels) or hematoxylin (bottom panels). Dark and light blue bars indicate the basal and suprabasal layers, respectively. Scale bar, 10 μm. (**D**) Quantification of the 5.8S rRNA immunoreactivity in the organotypic cultures displayed in (C). ***P < 0.0001 (ANOVA and Dunnett’s multiple comparison test, *n* = 5 independent cultures. In (**B**) and (**D**), data represent the mean ± SEM. Source data for this figure are provided as a Source Data file.

We next investigated whether ribosome biogenesis is upregulated in VAV2^Onc^-expressing primary keratinocytes and, if so, whether it contributes to the epithelial hyperplasia induced by this constitutively active protein. We used organotypic three dimensional (3D) cultures of primary mouse or human keratinocytes as the main working model, which allowed us to monitor the regulation of ribosome biogenesis in a tissue-like model that recapitulates all the differentiation stages of keratinocytes^11^. Using keratinocytes from either newborn wild-type (WT) or *Vav2*^Onc/Onc^ knock-in mice, we found that the cells with endogenous expression of VAV2^Onc^ generated thicker layers of suprabasal cells than their WT counterparts (Fig. 1A). These results are consistent with previous observations indicating that VAV2^Onc^ promotes the proliferative expansion of highly undifferentiated keratinocytes located in the suprabasal layer^11^. To assess ribogenesis activity in all cell layers of the epithelia, we stained sections from these 3D cultures with an antibody against 5.8S rRNA (5.8S), which is an integral component of the large 60S ribosome subunit that is generated from the 47S pre-rRNA precursor ^16^. We found that the epithelia generated by WT keratinocytes showed high levels of ribogenesis in the basal layer and, to a much lesser extent, the suprabasal cells (Fig. 1A). In contrast, the hyperplastic epithelia formed by *Vav2*^Onc/Onc^ keratinocytes displayed high levels of 5.8S rRNA immunoreactivity both in basal cells and in the highly expanded layer of suprabasal cells (Fig. 1A,B).

We next carried out similar analyses using primary human keratinocytes that stably expressed VAV2^Onc^ or VAV2^Onc+E200A^. The latter protein lacks enzyme activity due to the presence of an inactivating point mutation in the VAV2 catalytic domain^11^. As negative control, we used primary human keratinocytes expressing an empty lentivirus. As positive control, we utilized cell derivatives ectopically expressing c-MYC, a transcriptional factor involved in ribosome biogenesis^16^. The generation, validation, and biological characterization of these keratinocyte lines have been reported before^11^. As previously described^11^, we observed that keratinocytes expressing either VAV2^Onc^ or c-MYC promoted exacerbated levels of epithelial hyperplasia when tested in organotypic 3D cultures (Fig. 1C, upper panels). As for Vav2^Onc/Onc^ keratinocytes, this hyperplasia is the result of the expansion of highly proliferative and undifferentiated cells located in the suprabasal layer (Fig. 1C, second column of panels from left)^11^. In contrast, keratinocytes expressing VAV2^Onc+E200A^ generated epithelial structures similar to the control cells (Fig. 1C, third column of panels from left)^11^. This is consistent with the fact that the VAV2^Onc^-driven hyperplasia relies on a RHO GTPase-c-MYC signaling axis^11^. The staining of these sections with antibodies to the 5.8S rRNA revealed increased levels of 5.8S rRNA immunoreactivity in the expanded layers of suprabasal cells formed in the organotypic cultures of either VAV2^Onc^- or c-MYC-expressing keratinocytes (Fig. 1C,D). The stable expression of VAV2^Onc+E200A^ in keratinocytes did not change the usual pattern of 5.8S rRNA immunoreactivity found in the epithelial structures formed by the control cells (Fig. 1C,D), again indicating that the effects elicited by VAV2^Onc^ on the distribution of 5.8S rRNA immunoreactivity are catalysis dependent. Using similar organotypic 3D culture experiments, we demonstrated that the stable expression of constitutively active versions of RAC1 (F28L mutant), RHOA (F30L mutant), CDC42 (F28L mutant), or the combination of RAC1^F28L^ and RHOA^F30L^, promoted a redistribution of 5.8S rRNA immunoreactivity in the organotypic sections very similar to that seen in VAV2^Onc^- or c-MYC-expressing keratinocytes (Supplementary Fig. 2). These GTPase mutant versions are chronically active due to accelerated GDP/GTP exchange rates^24^. In line with previous results^11^, we found that the hyperplasic layer was significantly thicker in the organotypic cultures generated using human keratinocytes expressing RAC1^F28L^ + RHOA^F30L^ (Supplementary Fig. 2).

To further confirm the increased ribogenic activity in VAV2^Onc^-expressing keratinocytes, we next analyzed the number and size of the nucleoli present in our collection of human keratinocyte derivatives. Nucleoli are the nuclear structures in which rRNA is transcribed and initially processed, so their structure can undergo significant changes depending on the ribogenic activity of cells^16^. As compared to controls, we found that keratinocytes expressing VAV2^Onc^, c-MYC, or RAC1^F28L^ + RHOA^F30L^ had much larger nucleoli and a reduced average number of nucleoli present per cell (Supplementary Fig. 3A–C). Taken together, these results indicate that the constitutive activation of the catalytic-dependent pathways of VAV2 promotes enhanced ribogenesis in both mouse and human keratinocytes.

### VAV2^Onc^ promotes rRNA synthesis in an RNA polymerase I-dependent manner

**Figure 2:**
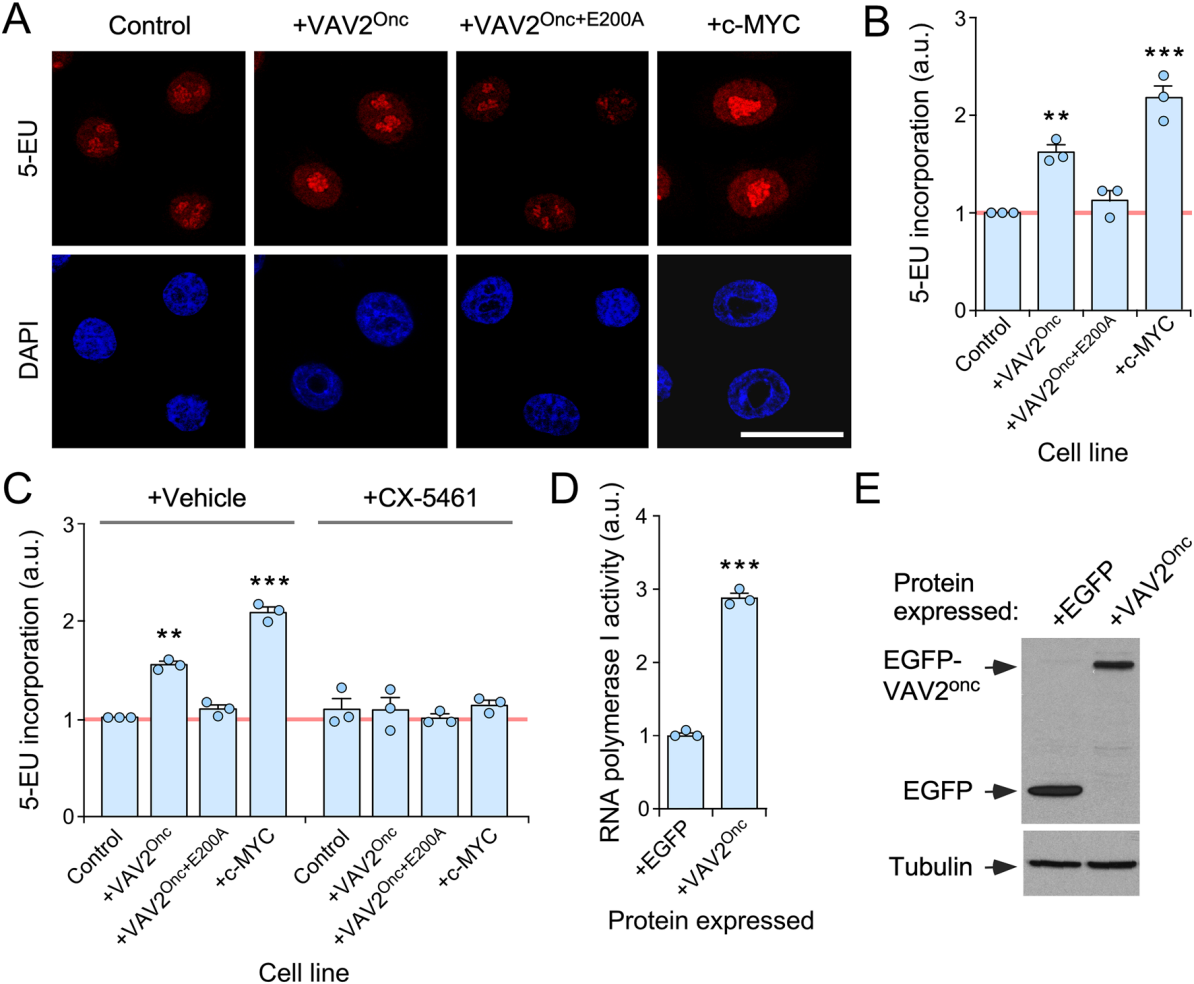
VAV2^Onc^-driven epidermal hyperplasia is associated with enhanced ribogenesis. (**A**) Representative images of 5-EU-labeled (red, top panels) and DAPI-labeled (blue, bottom panel) human keratinocytes expressing the indicated proteins (top). Scale bar, 20 μm. (**B**) Quantitation of the 5-EU incorporation in the experiments shown in (**A**). **, *P* = 0.003; ***, *P* < 0.0001 of indicated test samples versus control cells (ANOVA and Dunnett’s multiple comparison test, *n* = 3 independent experiments). a.u., arbitrary units. (**C**) Quantitation of 5-EU incorporation in the indicated keratinocyte lines after inhibition of RNA polymerase I with CX-5461. Each point represents the mean fluorescence intensity of an independent experiment (*n* = 50 cells scored in each case). **, *P* = 0.006; ***, *P* < 0.0001 (experimental test vs controls) (ANOVA and Tukey’s HSD tests, *n* = 3 independent experiments). (**D**) Determination of RNA polymerase I activity of keratinocytes transiently expressing the indicated proteins using a luciferase reporter assay. Values were normalized to the experimental value obtained in EGFP-expressing cells (which was given an arbitrary value of 1). *, *P* = 0.017; ***, *P* < 0.0001 (ANOVA and Tukey’s HSD tests, *n* = 3 biological replicates). (**E**) Immunoblot showing the expression of the ectopically expressed proteins in one of the experiments performed in (**D**) (top panel). Tubulin α was used as protein loading control (bottom panel). Similar results were obtained in two independent experiments (not shown). In (**B**) to (**D**), data represent the mean ± SEM. Source data for this figure are provided as a Source Data file.

To investigate how VAV2^Onc^ promotes enhanced levels of ribosome biogenesis, we first analyzed its impact on the synthesis of the primary 47S pre-rRNA precursor. To this end, we used pulse chase experiments with 5-ethynyl uridine (5-EU) followed by click chemistry-based reactions with an Alexa Fluor™ 594 carboxamido-(6-azidohexanyl) bis(triethylammonium) salt to label the nascent 47S pre-RNA precursors in exponentially growing 2D cultures of control, VAV2^Onc^-, VAV2^Onc+E200A^-, or c-MYC-expressing keratinocytes. We found that VAV2^Onc^-expressing cells and, to a larger extent c-MYC-expressing cells, displayed higher levels of 5-EU incorporation than control cells (Fig. 2A–C). In contrast, keratinocytes expressing the catalytically-dead VAV2^Onc+E200A^ protein displayed levels of 5-EU incorporation similar to the control cells (Fig. 2A–C). The addition of an RNA polymerase I inhibitor (CX-5461) to the cell cultures reduced the levels of 5-EU incorporation of cells expressing VAV2^Onc^ or c-MYC back down to those observed in control cells (Fig. 2C). These results suggest that the elevated levels of 47S pre-RNA production in VAV2^Onc^- or c-MYC-expressing keratinocytes is due to the activation of RNA polymerase I. This is likely the result of direct signaling, as we observed using luciferase reporter assays that VAV2^Onc^ can also promote RNA polymerase I activity when transiently transfected in primary human keratinocytes growing in 2D cultures (Fig. 2D,E).

We did not find any statistically significant changes in the abundance of pre-rRNA intermediaries that participate in the maturation stages of either the small (18S rRNA containing) or the large (28S rRNA containing) ribosome subunits in any of the interrogated keratinocyte lines (Supplementary Fig. 4). These findings indicate that VAV2^Onc^ primarily affects the RNA polymerase I-mediated synthesis of 47S pre-rRNA precursors in a catalytically-dependent manner.

### Mechanistic analysis of VAV2^Onc^-driven ribogenesis

**Figure 3:**
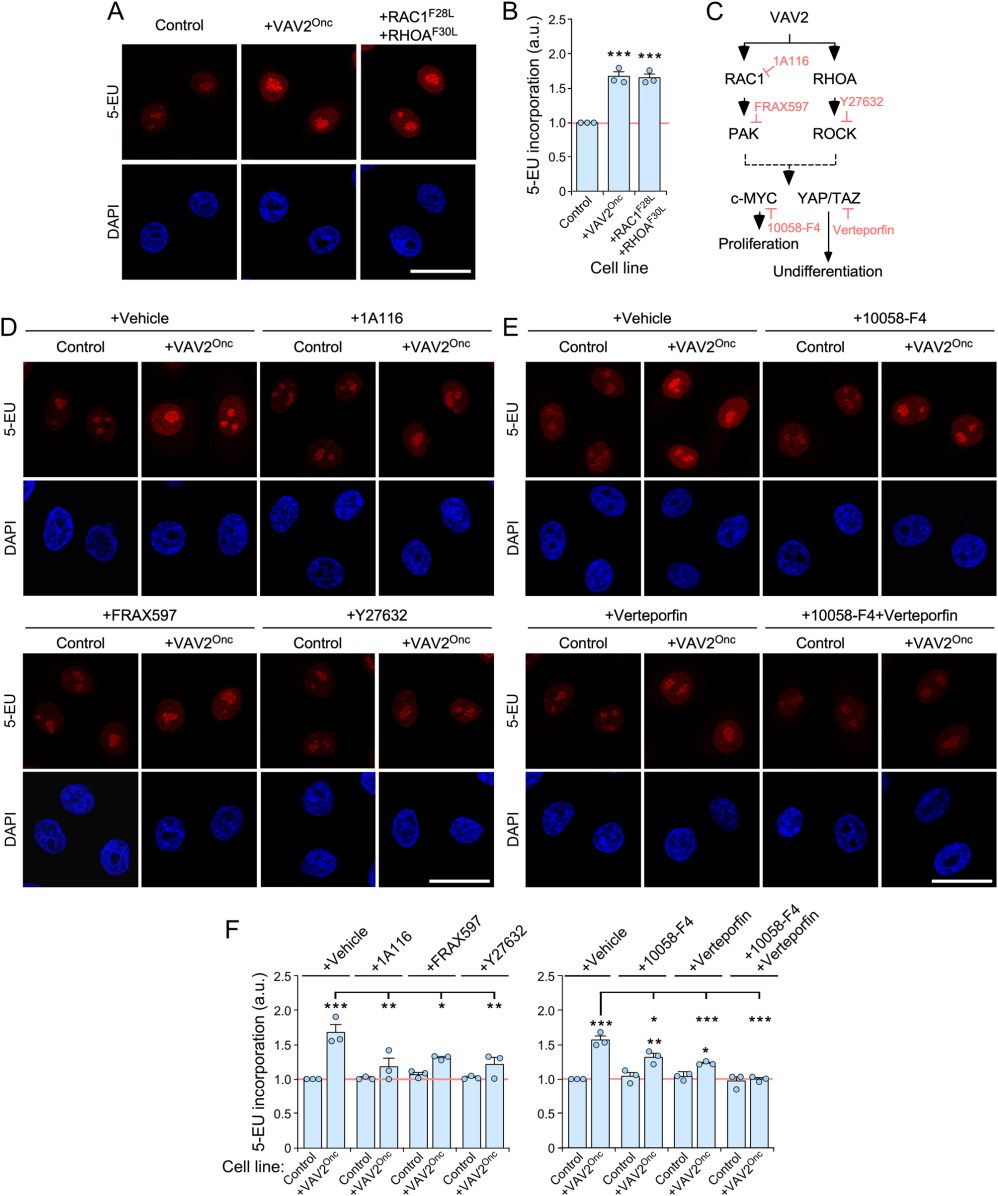
Mechanistic analysis of VAV2^Onc^-induced ribogenesis. (**A**) Representative images of 5-EU-labeled (red, top panels) or DAPI-labeled (blue, bottom panel) human keratinocytes expressing the indicated proteins (top). Scale bar, 20 μm. (**B**) Quantification of the incorporation of 5-EU from the experiments shown in (**A**). ***, *P* < 0.0001 (ANOVA and Dunnett’s multiple comparison tests, *n* = 3 independent experiments). (**C**) Schematic representation of the VAV2^Onc^-mediated regulation of cell proliferation and undifferentiation in human keratinocytes based on previous work^11^. Inhibitors targeting specific VAV2 downstream elements are shown in light red. (**D** and** E**) Representative images of 5-EU-labeled (red) or DAPI-labeled (blue) human keratinocytes expressing the indicated proteins and subjected to the culture conditions shown on the top. Scale bar, 20 μm. (**F**) Quantification of the incorporation of 5-EU from the experiments shown in (**D**) and (**E**). *, *P* < 0.05; **, *P* < 0.001; ***, *P* < 0.0001 vs controls or between the indicated experimental pairs (brackets) using ANOVA plus Tukey’s HSD tests. *n* = 3 independent experiments. In (B) and (F), data represent the mean ± SEM. Source data for this figure are provided as a Source Data file. Each point represents the mean fluorescence intensity of an independent experiment (*n* = 50 cells scored in each case).

We used the 5-EU labeling method described above to obtain further mechanistic information about how VAV2^Onc^ promotes ribosome biogenesis in keratinocytes. Consistent with it being a VAV2 catalysis-mediated mechanism (see Figs. 1 and 2), we found that keratinocytes ectopically expressing active RAC1^F28L^ + RHOA^F30L^ showed increased rates of 5-EU incorporation that were similar to those induced by VAV2^Onc^ (Fig. 3A,B). Next, we tested inhibitors of downstream elements of the VAV2^Onc^ pathway that are important for maintaining the regenerative proliferation in both mouse and human primary keratinocytes^11^ (Fig. 3C). These compounds target RAC1 (1A116), PAK (FRAX597), ROCK (Y27632), c-MYC (10058-F4), or the YAP/TAZ complex (verteporfin) (Fig. 3C). It is worth noting that we selected for these experiments concentrations of drugs that did not affect ribosome biosynthesis in control cells (see Materials and Methods). With this approach, we could circumvent the expected lethal effect of the blockage of this essential biosynthetic program in the interrogated cells. In addition, this strategy allowed us to identify therapeutic vulnerabilities that could be specific for VAV2^Onc^- and/or RAC1^F28L^ + RHOA^F30L^-expressing cells. While each of those inhibitors eliminated the high levels of 5-EU-labeled precursors when added to VAV2^Onc^-expressing cells, none of them elicited any statistically significant changes in the basal levels of 5-EU incorporation of control cells (Fig. 3D–F).

**Figure 4:**
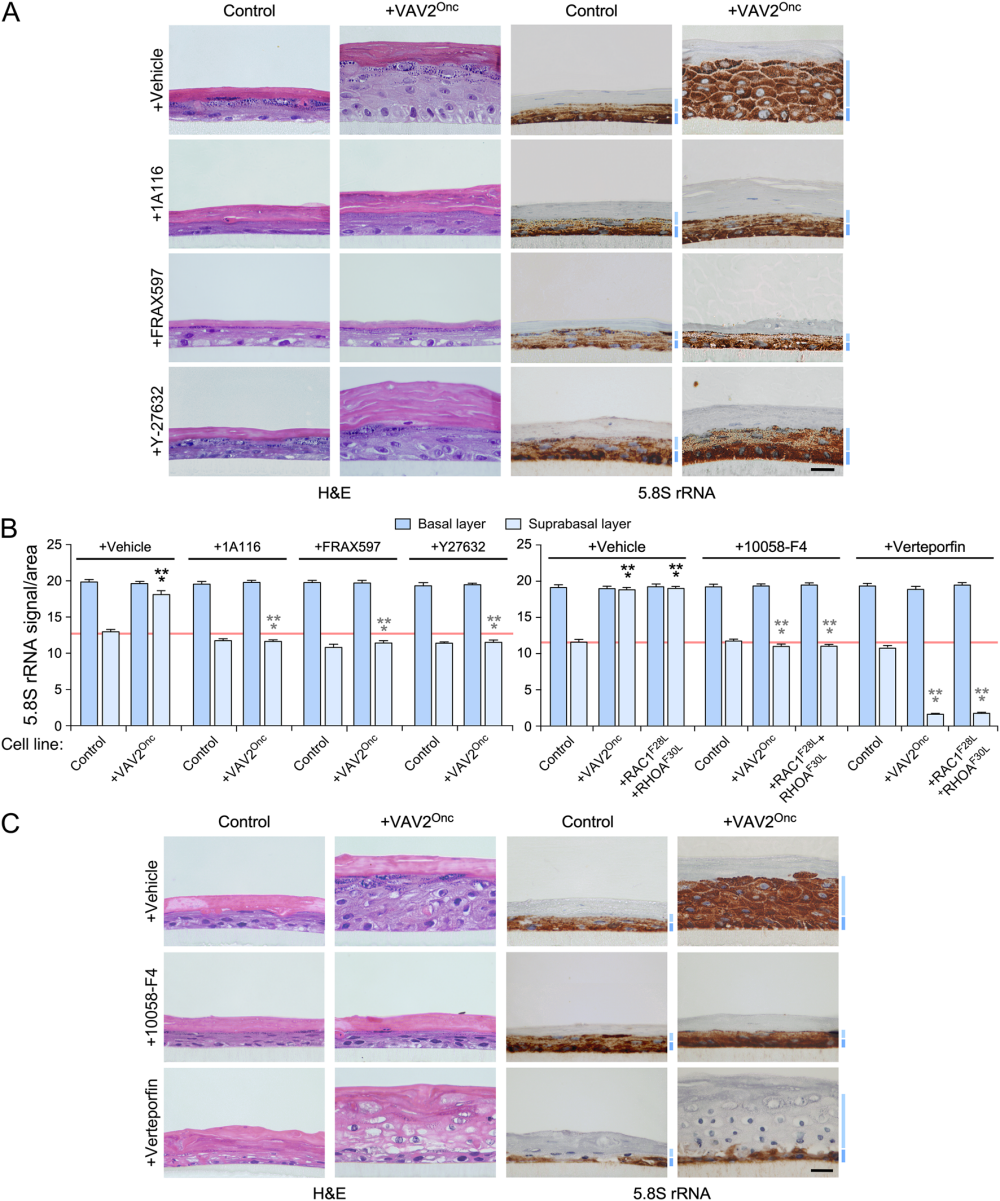
VAV2^Onc^-driven ribogenesis requires PAK, ROCK, MYC, and YAP/TAZ. (**A** and** C**) Representative images of organotypic cultures of human keratinocytes expressing the indicated proteins (top) after staining with either H&E (two left columns; A and C) or labeled with an antibody to the 5.8S rRNA plus hematoxylin (two right panels; **A** and **C**). Dark and light blue bars represent the 5.8S rRNA immunoreactivity levels found in the basal and suprabasal epithelial layers, respectively. Scale bar, 10 μm. (**B**) Quantitation of the 5.8S rRNA immunoreactivity obtained in panels (**A**) and (**C**) of this figure as well as in the experiments shown in Supplementary Fig. 5 (right panel). Black and gray asterisks indicate the *P* value of the indicated experimental values in untreated and treated cells when compared to the appropriate controls. ***, *P* < 0.0001 (ANOVA and Tukey’s HSD tests, *n* = 3 independent experiments). Data represent the mean ± SEM. Source data for this figure are provided as a Source Data file.

We have previously shown using organotypic 3D cultures that the addition of the inhibitors for RAC1, PAK, ROCK, or c-MYC blocks the epidermal hyperplasia induced by the stable expression of VAV2^Onc^ in keratinocytes^11^ (see example in Fig. 4A). We found that this process is associated with a change in the distribution pattern of the 5.8S rRNA, as the inhibitor-treated 3D cultures of VAV2^Onc^-expressing keratinocytes show a control-like 5.8S rRNA immunoreactivity pattern (Fig. 4A; see quantitation in Fig. 4B, left). The same effect was observed in the organotypic cultures generated by VAV2^Onc^-expressing cells treated with c-MYC inhibitors (Fig. 4C; see quantitation in Fig. 4B, right). Unlike the rest of inhibitors, the addition of the YAP-TEAD complex inhibitor verteporfin promotes an extensive differentiation of keratinocytes located in the suprabasal layer^11^. As a consequence, sections from these organotypic cultures exhibited highly enlarged suprabasal layers that, in this case, were mostly composed of differentiated cells^11^ (Fig. 4C). The 5.8S rRNA immunoreactivity was totally absent from that differentiated layer (Fig. 4C; see quantitation in Fig. 4B, right). The same results were obtained in organotypic cultures of keratinocytes expressing RAC1^F28L^ + RHOA^F30L^ treated with a c-MYC or YAP/TAZ inhibitor (Supplementary Fig. 5; for quantitation, see Fig. 4B, right). Taken collectively, these results indicate that the upregulation of ribosome biogenesis is integrated into the same VAV2^Onc^-regulated signaling framework that promotes regenerative proliferation. They also indicate that ribogenesis is dually influenced by two independent mechanisms: (i) the c-MYC- and YAP/TAZ-dependent effects on RNA polymerase I activity, and (ii) the YAP/TAZ-mediated blockage of cell differentiation that favors a cell state intrinsically associated with high ribogenesis rates.

### Ribogenesis contributes to VAV2^Onc^-driven epidermal hyperplasia

**Figure 5:**
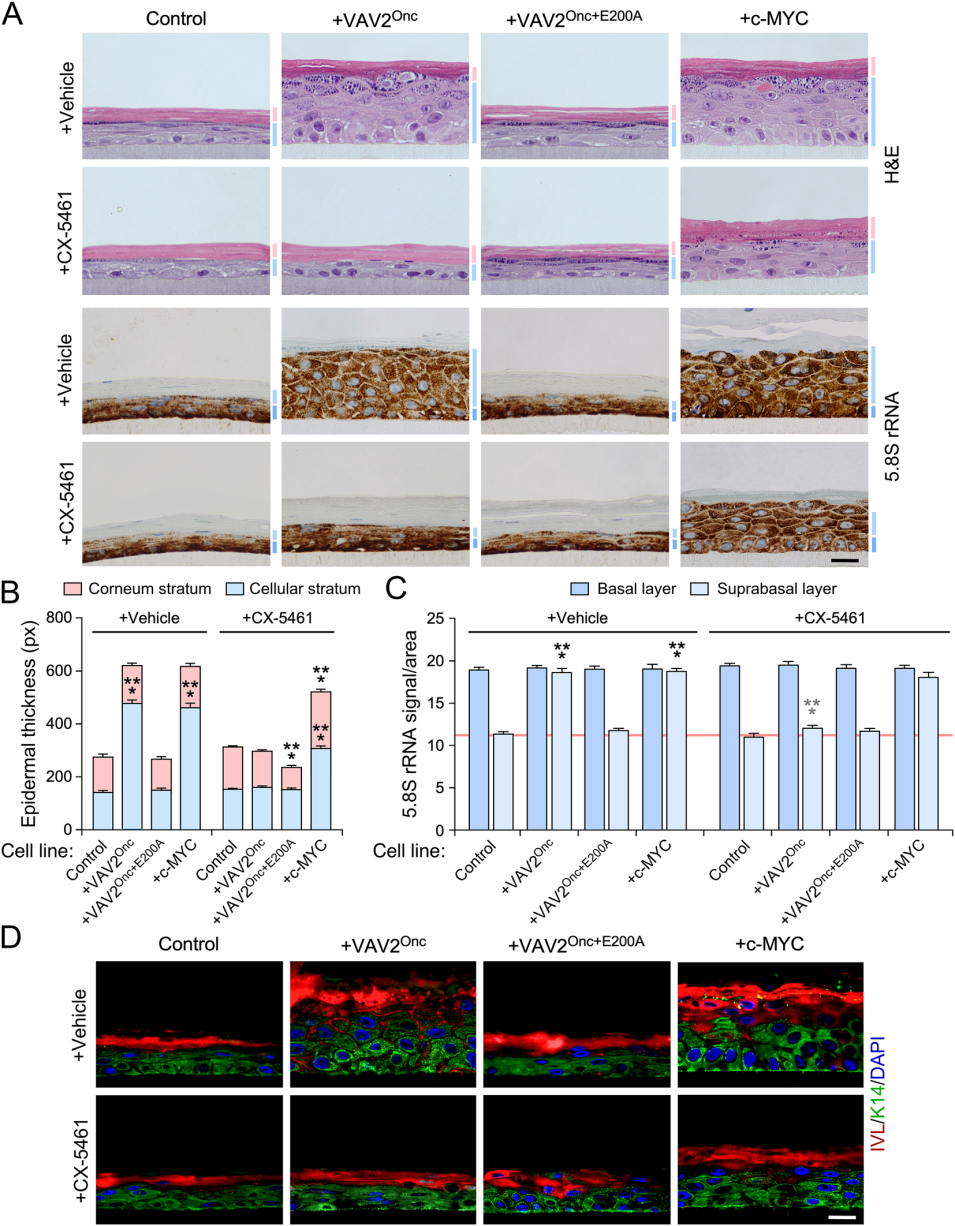
Ribogenesis is important for VAV2^Onc^-driven epidermal hyperplasia. (**A**) Representative images of organotypic cultures of human keratinocytes expressing the indicated proteins (top) and subjected to the culture conditions shown on the left. Cells were stained with either H&E (two top rows of panels) or labeled with an antibody to the 5.8S rRNA plus hematoxylin (two bottom rows of panels). Dark and light blue bars represent the 5.8S rRNA immunoreactivity levels found in the basal and suprabasal epithelial layers, respectively. Scale bar, 10 μm. (**B** and** C**) Quantification of the thickness (**B**) and 5.8S rRNA immunoreactivity (**C**) of indicated cell layers obtained in the experiments shown in (**A**). In (**C**), black and gray asterisks indicate the *P* value of the indicated experimental values as compared to control or vehicle-treated cells, respectively. ***, *P* < 0.0001 (ANOVA and Dunnett’s multiple comparison tests, *n* = 3 independent cultures). Data represent the mean ± SEM. Source data for this figure are provided as a Source Data file. px, pixel. (**D**) Representative images showing the expression of involucrin (IVL, red) and keratin 14 (K14, green) in organotypic cultures from the indicated human keratinocytes (top) and culture conditions (left). Nuclei were counterstaining with DAPI (blue color). Scale bar, 10 μm. Similar results were obtained in two additional independent experiments (not shown).

Given that the concentrations of inhibitors used in the previous experiments did not have any negative effects on the ribosome ribogenesis of control cells, we hypothesized that keratinocytes with upregulated VAV2 signaling could be highly dependent on high ribosome biogenesis rates to promote hyperplasia in organotypic cultures. To test this idea, we investigated the effects of the CX-5461 inhibitor of RNA polymerase I on the organotypic cultures generated by keratinocytes expressing VAV2^Onc^ or c-MYC. Given the lethality associated with the total blockage of this polymerase, we selected a concentration of CX-5461 that did not impair the growth of either control or VAV2^Onc+E200A^-expressing cells (Fig. 5A–C). CX-4561 eliminated the hyperplasia (Fig. 5A,B) and restored a control cell-like distribution of the 5.8S rRNA in the epithelia formed by VAV2^Onc^-expressing keratinocytes (Fig. 5A,C). This result indicates that the tissue hyperplasia generated by these cells is highly dependent on high ribogenesis rates. In contrast, CX-4561 was much less effective when tested in 3D cultures of c-MYC-expressing cells (Fig. 5A,B). This is probably due to the higher rates of ribosome biogenesis in those cells, as inferred from the high levels of 5.8S rRNA immunoreactivity that was still detected in the CX-5461-treated epithelial structures formed by them (Fig. 5A,C). This idea is also consistent with the higher levels of 5-EU incorporation exhibited by these cells as compared to VAV2^Onc^-expressing keratinocytes (see above, Fig. 2B,C).

In contrast to the cell differentiation effect triggered by verteporfin (see above, Fig. 4C)^11^, the treatment of VAV2^Onc^-expressing cells with the CX-5461 inhibitor did not induce any histological signs of differentiation in the organotypic cultures (Fig. 5A). This suggests that this inhibitor affects the proliferation rather than the differentiation of those cells. In line with this, we also observed that the distribution of cells positive for involucrin (a marker for differentiated cells) or for keratin 14 (a marker for undifferentiated cells) was indistinguishable in the sections obtained from organotypic cultures generated by control cells, VAV2^Onc+E200A^-expressing cells, or VAV2^Onc^-expressing cells treated with CX-5461 (Fig. 5D).

### The endogenous VAV2 pathway influences ribogenesis in OSCC cells

**Figure 6:**
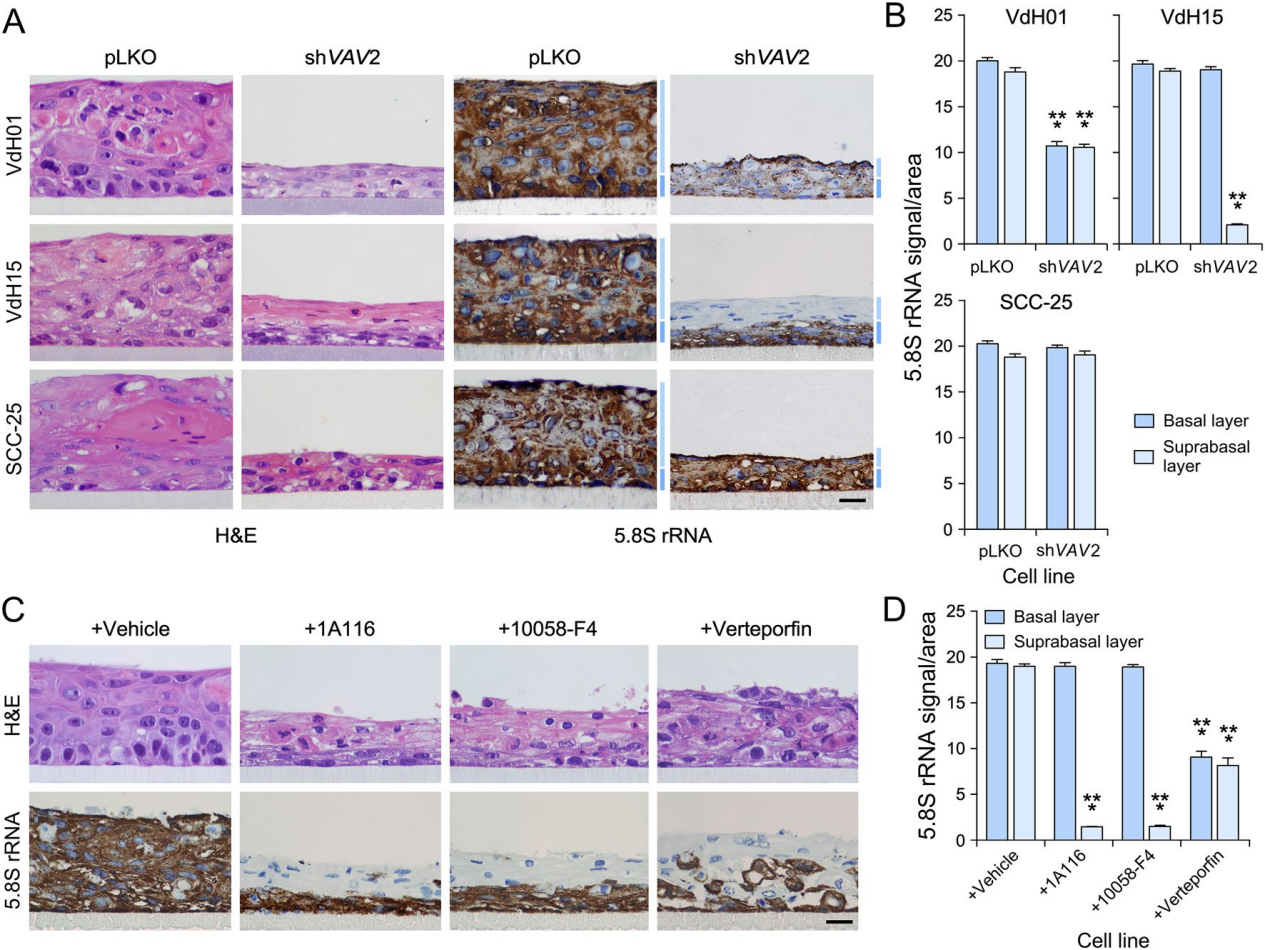
The endogenous VAV2 pathway influences ribogenesis in OSCC cells. (**A**) Representative images of organotypic cultures of indicated control and *VAV2*-knockdown OSCC cells that were stained with either H&E (two left panels) or labeled with an antibody to the 5.8S rRNA plus hematoxylin (two right panels). Dark and light blue bars (right) indicate cell layers analogous to the basal and suprabasal strata formed by normal keratinocytes, respectively. Scale bar, 10 μm. pLKO, control cells containing an empty lentiviral vector. (**B**) Quantitation of the 5.8S rRNA immunoreactivity obtained in panel (A). ***, *P* < 0.0001 (Student’s t-test, *n* = 3 independent experiments). (**C**) Representative images of organotypic cultures of VdH15 cells treated as indicated (top) that were stained with either H&E (top panels) or labeled with an antibody to the 5.8S rRNA plus hematoxylin (bottom panels). Scale bar, 10 μm. (**D**) Quantitation of the 5.8S rRNA immunoreactivity obtained in panel (**C**). ***, *P* < 0.0001 (Student’s t-test, *n* = 3 independent experiments). In (**B**) and (**D**), data represent the mean ± SEM. Source data for this figure are provided as a Source Data file.

Having established that VAV2 signaling positively influences ribosome biogenesis rates in normal keratinocytes, we next investigated whether the activity of endogenous VAV2 is also important for maintaining the ribogenic activity of already transformed OSCC cells. To this end, we used two previously described PDCs from distinct HPV^−^ OSCC patients (VdH01, VdH15) that were stably transduced with control or *VAV2* short hairpin RNA (shRNA)-encoding lentiviral particles^11^. In addition, we included control and *VAV2* knockdown derivatives of an OSCC cell line (SCC-25) that were generated following a similar strategy^11^. We previously demonstrated that the endogenous WT VAV2 protein is important for maintaining both the high proliferation and undifferentiated features of these three cell lines^11^ (see example, Fig. 6A). Staining sections of the tissue structures formed by these cells in 3D cultures using 5.8S rRNA antibodies revealed that VdH01 and VdH15 cells are also highly dependent on endogenous VAV2 for maintaining high rates of ribosome biogenesis (Fig. 6A,B). The SCC-25 cell line, although highly dependent on VAV2 for overall growth (Fig. 6A)^11^, maintained similar levels of the 5.8S rRNA in the absence or presence of endogenous VAV2 (Fig. 6A,B). The pathway that contributes to ribogenesis in OSCC PDCs was the same as the one in normal keratinocytes (see above, Figs. 3, 4, 5), as we observed that the inhibitors for RAC1, c-MYC or YAP/TAZ reduced the 5.8S rRNA immunoreactivity to levels similar to those found in *VAV2* knockdown cells (Fig. 6C,D). These results indicate that the PDCs interrogated in this study are highly dependent on the activity of endogenous VAV2 signaling for maintaining optimal rates of ribosome biogenesis. Moreover, the data obtained with SCC-25 cells suggest that, in some cases, the proliferative and ribogenic activity of cancer cells can independently be regulated by VAV2-dependent and independent pathways, respectively.

### Ribosome ribogenesis is a therapeutic Achilles’ heel for VAV2-dependent OSCCs

**Figure 7:**
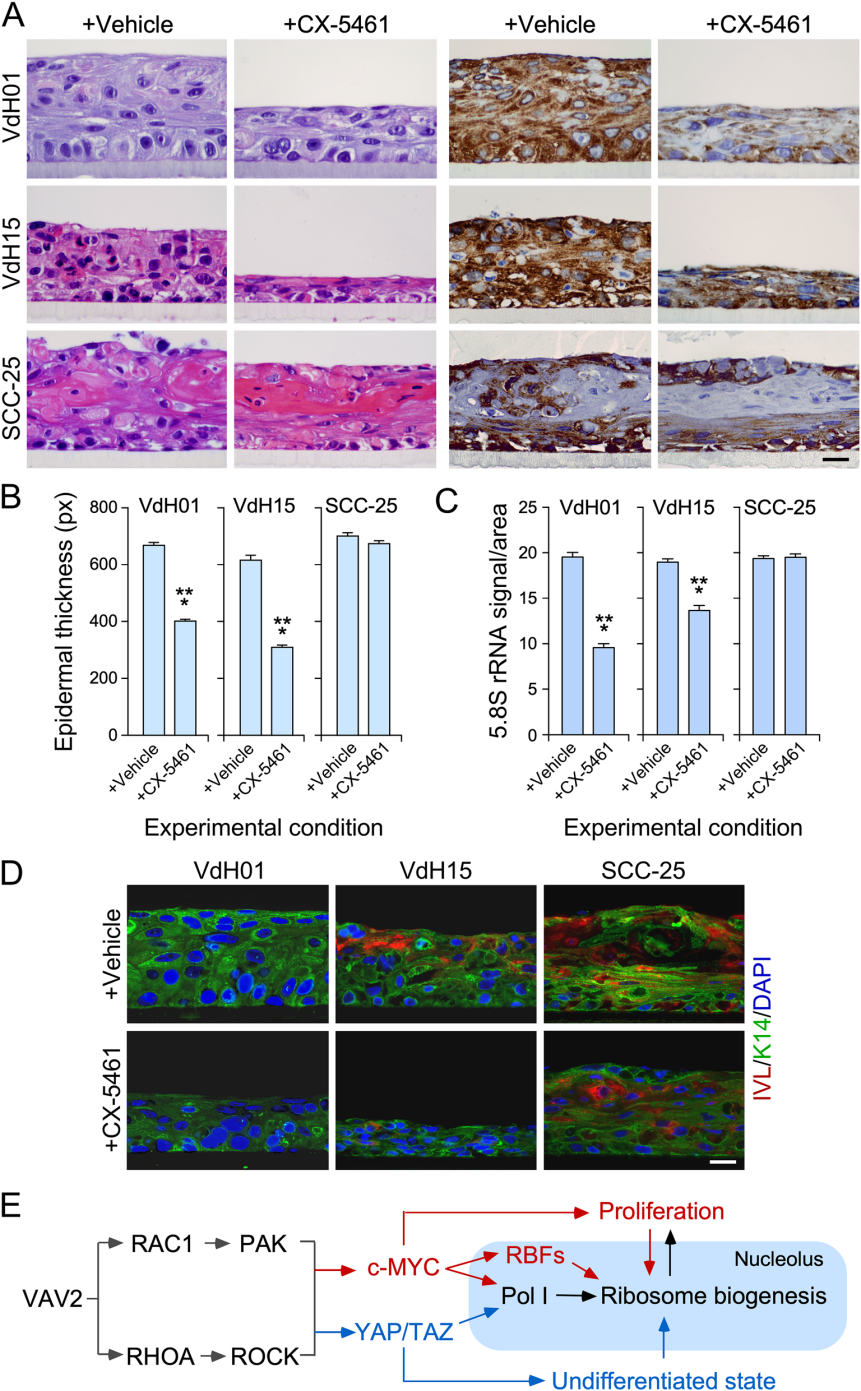
OSCC cells are sensitive to high rates of ribosome ribogenesis. (**A**) Representative images of organotypic cultures of indicated OSCC cells (left) that were treated with either vehicle solution or CX-5461 (top). Sections were stained with H&E (two left columns) or labeled with an antibody to the 5.8S rRNA plus hematoxylin (two right columns). Scale bar, 10 μm. (**B** and **C**) Quantitation of the thickness of the epithelium (**B**) and the 5.8S rRNA immunoreactivity (**C**) from the experiments shown in (**A**). ***, *P* < 0.001 (Student’s *t*-test, *n* = 3 independent experiments). Data represent the mean ± SEM. Source data for this figure are provided as a Source Data file. (**D**) Representative images of organotypic models from indicated OSCC cell lines (top) and culture conditions (left) that were stained with antibodies to either involucrin (IVL, red) or keratin 14 (K14, green) and counterstained with DAPI (blue). Scale bar, 10 μm. Similar results were obtained in two additional experiments (not shown). (**E**) Proposed model for the regulation of ribosome biogenesis by the VAV2 catalysis-dependent pathways in normal and transformed keratinocytes. Pol I, RNA polymerase I; RBFs, ribosome biogenesis factors. The nucleolus is shaded in blue. The bidirectional arrows between proliferation and ribosome biogenesis indicate that both processes influence each other. The unidirectional arrow between the undifferentiated state and ribosome biogenesis indicates that the former affects the latter but not vice versa.

Previous reports have shown that the elimination of specific ribosome biogenesis factors (HEATR1, NOB1, PES1, RIOK2) impairs the proliferation and malignant traits of OSCC cell lines^25–28^. This is not entirely surprising given that the elimination of these proteins is lethal^17^. Likewise, it has been shown that the inhibition of RNA polymerase I per se or in combination with mTOR reduces the in vivo tumorigenicity of an OSCC cell line using orthotopic xenotransplant experiments^15^. To further assess this issue, we investigated the effects of CX-5461 on VdH01, VdH15, and SCC-25 cells using organotypic 3D cultures. Again, we selected a concentration of the inhibitor that did not impair the growth of normal keratinocytes to avoid the lethal effects caused by the total shutdown of this essential process. The RNA polymerase I inhibitor CX-5461 reduced the growth of the two OSCC PDCs used in our study (Fig. 7A,B). As expected, this process was also associated with a reduction in 5.8S rRNA immunoreactivity levels in both cases (Fig. 7A,C). In contrast, CX-5461 did not elicit any statistically significant effects on the 3D growth of SCC-25 cells (Fig. 7A,B) and in 5.8S rRNA immunoreactivity (Fig. 7A,C), suggesting that this cell line might have higher RNA polymerase I activity than the two PDCs used in this study. Finally, we did not find any overt signs of differentiation in the sections obtained from CX-5461-treated PDC 3D cultures (Fig. 7D).

## Discussion

There is still very scant information on the signaling connections established between RHO GTPases and ribogenesis in either normal or cancer cells. In this work, we have demonstrated that the RHO GEF VAV2 plays important roles in the regulation and maintenance of high ribosome biogenesis rates in normal keratinocytes and OSCC PDCs, respectively. This result indicates that the correlation seen in bioinformatics analyses between the expression levels of the *VAV2* mRNA and the ribosome biogenesis-related gene signatures in patient samples probably reflects a direct functional connection between those two routes rather than just a mere statistical correlation.

Mechanistic analyses performed using both 2D and 3D cultures indicate that, unlike the ribogenesis-linked functions of both ECT2 and ARHGAP30^21,22,29^, the connection established between VAV2 and ribogenesis involves a more canonical signaling pathway that entails the VAV2-mediated stimulation of the GTPases RAC1 and RHOA, the proximal effectors PAK and ROCK, and the transcriptional factors c-MYC and YAP/TAZ. The stimulation of both c-MYC and YAP/TAZ leads to increased production of the 47S pre-rRNA precursor in the nucleolus via increased RNA polymerase I activity (Fig. 7E). In addition, we believe that the YAP/TAZ complex also contributes indirectly to this process by maintaining a highly undifferentiated cell state that is compatible with full ribogenic activity (Fig. 7E). It is likely that these two inputs are further boosted by the large collection of ribosome biogenesis factors that become upregulated during VAV2^Onc^-driven epidermal hyperplasia. Although we have not mechanistically dissected this part of the equation, previous studies suggest that this could be mediated by c-MYC^16^ (Fig. 7E). Given the level of conservation of this route in primary keratinocytes and OSCC PDCs, it is likely that the mechanism reported here for the VAV2-mediated regulation of ribosomal biogenesis will be also operative in other subtypes of HNSCC.

Interestingly, the regulation of ribosome biogenesis in basal cells seems to be different from the mechanistic model shown here for the hyperplasic keratinocytes (Fig. 7E). This is based on several observations: (i) the 5.8S rRNA immunoreactivity is mostly concentrated in the basal layer of the epithelial structures formed by control cells; (ii) this immunoreactivity is preserved in organoid cultures from VAV2^Onc^-expressing or RAC1^F28L^ + RHOA^F30L^-expressing keratinocytes treated with inhibitors for downstream VAV2 signaling elements; and (iii) the 5.8S rRNA immunoreactivity is also maintained after the CX-5461 treatment of 3D cultures from control keratinocytes, VAV2^Onc^-expressing keratinocytes, or RAC1^F28L^ + RHOA^F30L^-expressing keratinocytes. This latter result suggests that basal cells in all of these cultures have higher levels of RNA polymerase I activity than their suprabasal counterparts. The VAV2-independent mechanism that controls ribogenesis in basal cells remains to be determined. However, it is likely that it is also ECT2- and RAC1-independent, given that the RAC1 inhibitor used in our study did not affect the 5.8S rRNA immunoreactivity levels of these cells.

VAV2 signaling plays critical roles in the regenerative proliferation of keratinocytes, a feature that is associated with poor prognosis of patients^11^. We surmise therefore that targeting the signaling elements of this pathway can be potentially used to treat this type of tumors. The results presented here further emphasize this idea, as demonstrated by the negative effects of the CX-5461 inhibitor on the growth of PDCs in 3D cultures. We did not observe any changes in these parameters in control cells at the concentrations of inhibitors used, suggesting that there could be therapeutic windows in which the treatments with these drugs will not interfere with the fitness of healthy cells in patients. Interestingly, we observed that the dependency on ribosome biogenesis can vary depending on the OSCC cell type used. For example, we found that ribogenesis is both VAV2- and CX-5461-independent in the SCC-25 cell line. The growth of SCC-25 cells is VAV2-dependent^11^, suggesting that these cells have acquired specific signaling and/or genetic alterations that have uncoupled the regulation of ribogenesis from the rest of VAV2-dependent processes.

How can this VAV2-regulated process be targeted in OSCC and other HNSCC subtypes? One possibility is to target VAV2 itself via either standard or PROTAC-based inhibitory approaches. However, this strategy has been a problematic so far for other RHO GEFs^30^. Another strategy is to focus on more druggable downstream elements such as c-MYC or the YAP/TAZ complex^31–33^ that, currently, are being tested in clinical trials (NCT05100251, NCT05228015, NCT04665206). This option has the advantage that it can kill regenerative proliferation and ribogenesis at the same time in tumors. Another plausible option is to target the RNA polymerase I itself (Fig. 7E), an avenue that has already been demonstrated to be effective in mouse models^15,34,35^ and is being currently tested in clinical trials (NCT04890613). Further studies will be needed to pinpoint the best therapeutic strategies to block this pathway in HNSCC and, perhaps, in other VAV2-dependent SCC subtypes.

## Materials and methods

### Ethics statement

All animal work was performed in accordance with protocols approved by the Bioethics committee of Salamanca University and the animal experimentation authorities of the autonomous government of Castilla y León (Spain). The part of our study involving animal work is reported in accordance with ARRIVE guidelines. The use of PDCs was conducted according to methods and a priori informed patient consent policies approved by the Bioethics committees of the Vall d’Hebron Research Institute. All experiments conducted in this work have been performed in accordance with relevant guidelines and regulations.

### Plasmids

The plasmid encoding EGFP-VAV2^Onc^ (pNM115) was described previously^11^. For its construction, the cDNA encoding VAV2^Onc^ (Δ1 − 186) was liberated from plasmid pKES19^12^ by digestion with BstXI, filled in, and cloned into the SmaI-linearized pEGFP-C2 vector (Clontech, cat. #632481). Luciferase activity was tested using a plasmid encoding luciferase under the regulation of the rRNA promoter (provided by L.-L. Chen, Institute of Biochemistry and Cell Biology, Chinese Academy of Sciences, University of Chinese Academy of Sciences, 200031 Shanghai, China) and pRL-SV40 (*Renilla* luciferase, obtained from Promega, cat. #E2231). The DNA sequences of all plasmids were verified.

### Cells

Primary human keratinocytes (Ker-CT cell line, immortalized by the ectopic expression of both TERT and CDK4) were obtained from the American Type Culture Collection (cat. #CRL-4048). These cells were cultured in CnT-Prime medium (CELLnTEC, cat. #CnT-PR) and transfected in KGM-Gold medium (Lonza, cat. #00192060). OSCC PDCs (VdH01, VdH15) were generously provided by S.A. Benitah (Institute for Research in Biomedicine, Barcelona, Spain) and have been described elsewhere^11,36^. VdH01 cells were cultured in FAD^+^ medium, which is a combination of 75% DMEM (Gibco, cat. #21969) and 25% Ham’s F-12 medium (Thermo Fisher, cat. #11765054) that was supplemented with 10% fetal bovine calf serum (Gibco, cat. #10270106), 2 mM L-glutamine (Gibco, cat. #25030024), 1.8 × 10^−4^ M adenine (Sigma-Aldrich, cat. #A2786-5G), 0.5 μg/mL hydrocortisone (Sigma-Aldrich, cat. #H4001-1G), 5 μg/mL insulin (Thermo Fisher, cat. #12585014), 10 ng/mL epidermal growth factor (PreproTech, cat. #AF-100-15) and 10^−10^ M cholera toxin (Sigma-Aldrich, cat. #C8052-5MG). VdH15 cells were grown in KSFM medium supplemented with 25 μg/mL BPE and 0.5 ng/ml epidermal growth factor. SCC-25 cells were provided by S.A. Benitah and cultured in KSFM medium supplemented with 25 μg/mL BPE and 0.5 ng/mL epidermal growth factor. Derivatives from all those cells expressing or lacking the indicated proteins were generated and validated in a previous study from our lab^11^. When appropriate, inhibitors for indicated signaling elements were used. Those included: 1A116 (500 nM)^37,38^, FRAX597 (5 nM, Selleckchem, cat. #S7271), Y-27632 (1 μM, Selleckchem, cat. #S1049), 10058-F4 (500 nM, Selleckchem, cat. #S7153), verteporfin (100 nM, Selleckchem, cat. #S1786), and CX-5461 (100 nM, Selleckchem, cat. #S2684). In all cases, we selected concentrations of inhibitors for performing these experiments that did not cause major dysfunctions in control cells. To this end, we performed pilot 2D or 3D experiments in which control and test cells were treated with increasing concentrations of the drugs (1, 5, 10, 50, 100, 500 and 1000 nM). Based on those results, we then selected the highest drug concentration that impaired the interrogated functions of the tested cells and that did not affect the behavior of the control counterparts.

### Mouse models

*Vav2*^Onc/Onc^ knock-in mice and appropriate controls have been described elsewhere^39^. Animals were kept in ventilated rooms in pathogen-free facility of the University of Salamanca under controlled temperature (23 °C), humidity (50%), and illumination (12-h-light/12-h-dark cycle) conditions.

### In silico analyses of mouse expression microarray data

The functional annotation of the VAV2^Onc^-dependent transcriptome was reported before using the Gene Expression Omnibus (GEO) dataset GSE124019 [https://www.ncbi.nlm.nih.gov/geo/query/acc.cgi?acc=GSE124019]^11^. Gene set enrichment analyses (https://www.gsea-msigdb.org/gsea/index.jsp) were performed using the same GEO dataset using gene set permutations (*n* = 1000) for the assessment of significance and signal-to-noise metric for ranking genes. Protein interaction networks were built using the Cytoscape software (https://cytoscape.org, National Resource for Network Biology). To evaluate the expression of the VAV2^Onc^ gene signature for ribosome biogenesis factors across normal, dysplastic and tumoral samples, the enrichment score was calculated using ssGSEAs (https://www.genepattern.org/modules/docs/ssGSEAProjection/4#gsc.tab=0). To this end, we used the GEO GSE30784 dataset (*n* = 229 samples) [https://www.ncbi.nlm.nih.gov/geo/query/acc.cgi?acc=GSE30784]^40^. This dataset lacks information on HPV status, although the percentage of HPV^−^ cases has been estimated to be in the 75% range^40^.

Overall survival analyses were performed through Kaplan–Meier estimates according to the expression level of indicated signatures using the GEO GSE41613 [https://www.ncbi.nlm.nih.gov/geo/query/acc.cgi?acc=GSE41213] dataset. This dataset was selected because it contained information on long-term survival, HPV status, and other clinical criteria of patients. It also contains a number of samples (*n* = 97 cases, all of them HPV^−^) that were compatible with proper statistical analyses^41^. The median of the expression distribution of the indicated gene signature was used to establish the low and high expression groups and, subsequently, the Mantel-Cox test was applied to statistically corroborate the differences seen between the two survival distributions. The survival scores for the *EGFR* mRNA, the *VAV2* mRNA, and other VAV2^Onc^-regulated gene signatures were calculated in a previous study using the same method and GEO dataset^11^.

### Isolation of primary mouse keratinocytes

This was done as previously described^11^. Briefly, the skin from euthanized neonatal mice of indicated genotypes was incubated with 250 units/mL dispase (Roche, cat. #04942078001) in KSFM medium (Thermo Fisher, cat. #17005-042) for 16 h at 4 °C to separate the epidermis from the dermis. The epidermis was then treated with accutase (CELLnTEC, cat. #CnT-Accutase-100) for 30 min at 37 °C to release the keratinocytes. The isolated cells were then cultured in KSFM medium supplemented with 20 nM CaCl_2_, 25 μg/mL BPE and 0.25 ng/mL EGF (Thermo Fisher, cat. #37000-015).

### Three-dimensional organotypic cultures

Mouse (5 × 10^5^ cells) and human (2 × 10^5^ cells) keratinocytes were seeded onto 12-mm diameter inserts (Millipore, cat. #PIHP01250) and cultured in CnT-Prime medium. After two days, the medium was replaced with CnT-PR 3D-Barrier (CellnTec, cat. #CnT-PR-3D); 16 h later, the airlift was performed according to the manufacturer’s instructions. 3D cultures were maintained in CnT-PR 3D-Barrier for 12 days and ultimately fixed in 4% paraformaldehyde to be processed for immunohistochemical analysis. During the final seven days of the culture, cells were treated with the appropriate vehicles and inhibitors, including 1A116 (500 nM), FRAX597 (5 nM), Y-27632 (1 μM), 10,058-F4 (500 nM), verteporfin (100 nM), and CX-5461 (100 nM). Concentration of inhibitors were chosen based on their minimal effect on control cells as indicated above.

### Histological and immunohistochemical studies

Tissue sections were either stained with hematoxylin–eosin (H&E) or exposed to Tris EDTA [pH 8.0] for heat-induced antigen unmasking and subsequent incubation for 40 min with the appropriate primary antibody to 5.8S rRNA (1:1500 dilution, Santa Cruz, cat. #sc-33678), involucrin (1:100 dilution, Sigma-Aldrich, cat. #I9018) or keratin 14 (1:300 dilution, Biolegend, cat. #905301). Immunohistochemical staining was carried out using a Ventana Discovery Ultra instrument (Roche, cat. #05987750001). For standard staining, the Discovery OmniMap anti-rabbit horse radish peroxidase detection system (Roche, cat. #760-4311) was used for detection as specified by the manufacturer. For immunofluorescent studies, sections were incubated for 1 h with appropriate secondary antibodies to either rabbit or mouse IgGs labeled with Alexa Fluor 488 (1:200 dilution, ThermoFisher, cat. #A21206) and Cy3 (1:200 dilution, Jackson ImmunoResearch, cat. #115-165-146). For staining of nuclei, sections were incubated with 4′,6-diamidino-2-phenylindole dihydrochloride (DAPI) (Sigma-Aldrich, cat. #D9542) for 5 min. Immunohistochemical signals were quantified using Fiji software.

### Determination of nucleolar parameters

Exponentially growing cells were seeded onto 10-mm glass coverslips previously treated with poly-L-lysine (Sigma-Aldrich, cat. #F8775). After 48 h, cells were fixed with 4% paraformaldehyde in phosphate-buffered saline solution for 15 min, and permeabilized with 0.25% Triton (Sigma-Aldrich, cat. #X100) in TBS-T [25 mM Tris–HCl (pH 8.0), 150 mM NaCl, 0.1% Tween-20 (Sigma-Aldrich, cat. #P7949)] for 10 min. Coverslips were then blocked with 2% bovine serum albumin (BSA) in TBS-T for 30 min and incubated with a primary antibody to nucleophosmin (1:50 dilution, Invitrogen, cat. #32-5200) in a moist chamber for 2 h. Next, cells were incubated with the corresponding secondary antibody (1:500, Invitrogen, cat. #A28175) for 30 min and stained with DAPI for 5 min to visualize the nuclei. Coverslips were mounted onto glass slides using Mowiol medium and images captured using a Leica TCS-SP8 microscope. The nucleolar areas were measured using the Fiji software.

### 5-EU incorporation assays

Newly synthesized rRNA was monitored using the Click-iT RNA Alexa Fluor 594 Imaging Kit (ThermoFisher, cat. #C10330). For this, keratinocytes were plated onto poly-l-lysine-coated coverslips, cultured for 48 h, and treated with 1 mM 5-ethynyl uridine (5-EU) (ThermoFisher, cat. #C10330) for 20 min. Cells were then fixed with 4% paraformaldehyde in phosphate-buffered saline solution for 15 min, permeabilized with 0.25% Triton X-100 in TBS-T for 10 min and blocked with 2% BSA in TBS-T for 30 min. Nascent rRNA was detected using Alexa Fluor 594 according to the manufacturer's protocol. After labeling, nucleoli were stained with DAPI for 5 min and, subsequently, the coverslips mounted on slides. When indicated, cells were treated with the corresponding vehicle solution and inhibitors for 24 h. The inhibitors used were FRAX597 (5 nM), Y-27632 2HCl (1 µM), 1A116 (500 nM), 10,058-F4 (500 nM), and verteporfin (200 nM). Concentrations of inhibitors were selected based on the induction of minor effect in the organotypic structures formed by control cells as indicated above. Cell images were acquired using Leica TCS-SP8 microscope, and the signal intensity was measured using the Fiji software.

### Luciferase assay for detection of DNA polymerase I activity

Exponentially growing cells were transiently transfected using Fu-GENE HD reagent (Promega, cat. #E2311) with: (i) 80 ng of the pRL-SV40 vector encoding the Renilla luciferase gene used as an internal control for transfection efficiency; (ii) 2 μg of the reporter plasmid containing the firefly luciferase gene under the regulation of the rRNA promoter; or (iii) 2 μg of the indicated EGFP-derived plasmids. After 36 h, cells were lysed with Passive Lysis Buffer (Promega, Catalog. No. E1960) and luciferase activity determined using the Dual Luciferase Assay System (Promega, cat. #E1960). In all cases, the ratio of the firefly luciferase/renilla luciferase activity was calculated and normalized according to the values obtained in controls.

### Western blot analyses

Exponentially growing cells were washed with chilled phosphate buffered saline solution and then lysed in RIPA buffer at 4 °C. Extracts were precleared by centrifugation at 13 200 rpm for 10 min at 4 °C, denatured by boiling in SDS-PAGE sample buffer, separated electrophoretically, and transferred onto nitrocellulose filters using the iBlot Dry Blotting System. Membranes were blocked as above and then incubated overnight at 4 °C with appropriate antibodies to GFP (1:1000 dilution; Clontech, cat. #632,381) and tubulin α (1:2000 dilution; Calbiochem, cat. #CP06). After three washes with TBS-T to eliminate the primary antibody, the immunoreacting bands were revealed using a standard chemiluminescent method (Thermo Fisher Scientific, cat. #32106).

### Northern blot analyses

Total RNAs were extracted using the TRIzol method (TRI reagent, Ambion, cat. #AM9738) and quantified using a NanoDrop Spectrophotometer. Northern blot analyses were carried out following standard procedures after separation of RNA samples in 1.2% agarose/formaldehyde gels^42^. The following sequences of the oligonucleotides were used as probes: 5′-CCT CGC CCT CCG GGC TCC GGG CTC CGT TAA TGA TC-3′ (forward, *5*′*-ITS1*), 5′-GAT CAT TAA CGG AGC CCG GAG CCC GGA GGG CGA GG-3′ (reverse, *5*′*-ITS1*), 5′-CTG CGA. GGG AAC CCC CAG CCG CGC A-3′ (forward, *ITS2*), 5′-TGC GCG GCT GGG GGT TCC CTC GCA G-3′ (reverse, *ITS2*). RNA levels were quantified using the Fiji software.

### Statistical analyses

Statistics were calculated using GraphPad Prism 8.0 (Dotmatics). The number of biological replicates (n), the type of statistical test applied, and the statistical significance for each experiment are indicated in the figure legends. Data normality was tested using the Shapiro–Wilk test. Parametric distributions were analyzed using Student’s *t*-test (when comparing two experimental groups), one-way ANOVA followed by either Dunnett’s tests (when comparing more than two experimental groups with a single control group), or Tukey’s HSD tests (when comparing more than two experimental groups with every other group). In all cases, values were considered significant when *P* ≤ 0.05. Data obtained are expressed as the mean ± SEM. Heatmaps were generated using the heatmap3 *R* package^43^.

### Supplementary Information

Supplementary Figures.

Supplementary Information 2.

## Data Availability

All relevant data are available from the corresponding author upon reasonable request. A Materials Transfer Agreement could be required in the case of potential commercial applications.
