# Supplementary material for: VAV2 orchestrates the interplay between regenerative proliferation and ribogenesis in both keratinocytes and oral squamous cell carcinoma
Source: Sci Rep. 2024 Feb 19;14:4060. doi: 10.1038/s41598-024-54808-0 (PMC10876654; doi:10.1038/s41598-024-54808-0)

Supplementary Information for

VAV2 ORCHESTRATES THE INTERPLAY BETWEEN REGENERATIVE PROLIFERATION AND RIBOGENESIS IN BOTH KERATINOCYTES AND ORAL SQUAMOUS CELL CARCINOMA

Natalia Fernández-Parejo, L. Francisco Lorenzo-Martín *et al*.

This PDF file includes:

Supplementary Figures 1 to 5 and legends (pages 2-8)

Images of the original blots shown in Fig. 2E (page 9) and Supplementary Fig. 4B (page 10)

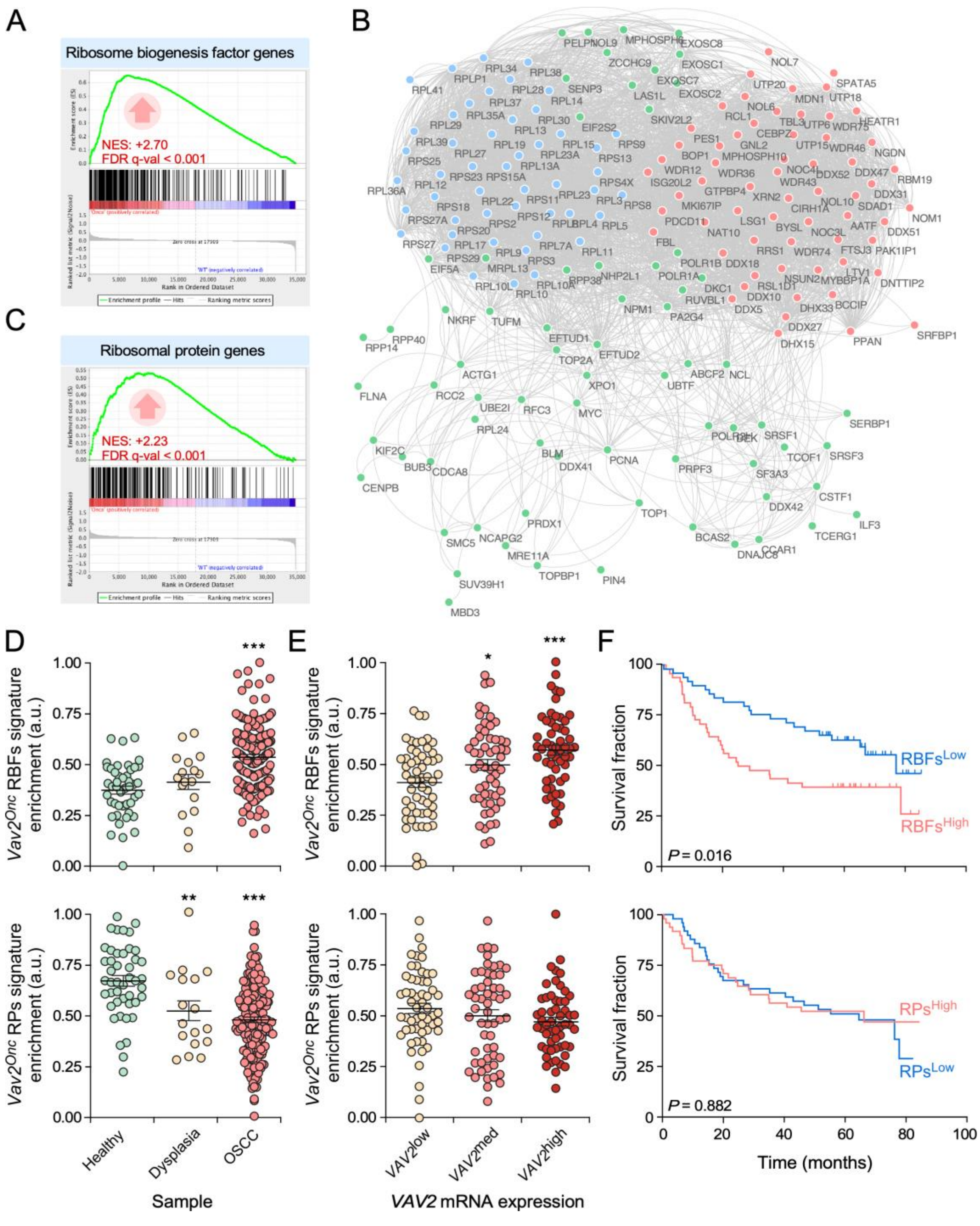

SUPPLEMENTARY FIGURE 1. Ribogenesis correlates with both VAV2 signaling and HNSCC patient prognosis

(A and **C)** GSEA enrichment plots showing the upregulation of gene sets for ribosome biogenesis factors (RBF, A) and ribosomal proteins (RP, B) in the VAV2^{Onc}-dependent transcriptome previously identified in the skin of mice. The normalized enrichment scores (NES) and false discovery rate q-values (FDR q-val) are indicated within each graph. Positive enrichments are indicated with upward arrows.

(B) Protein interaction network containing the 180 leading-edge genes obtained in (A) and (B). The red (right) and the blue (left) nodes constitute the $VAV2^{Onc}$ RBF and $VAV2^{Onc}$ RP signatures, respectively. The green nodes represent other regulators of ribosome biogenesis.

(D and **E)** Dot plots showing the enrichment of the gene signatures for $VAV2^{Onc}$-regulated RBF (D and E, top panels) and $VAV2^{Onc}$-regulated RP (D and E, bottom panels) according to patient sample (D) and *VAV2* mRNA expression levels within OSCC tumors (E). *, $P < 0.05$; **, $P < 0.01$; ***, $P < 0.001$ (ANOVA and Dunnett's multiple comparison tests). Data represent the mean ± SEM. Source data for this figure are provided as a Source Data file.

(F) Kaplan-Meier plots showing the survival rates of OSCC patients who were stratified according to the high and low expression levels of the signatures for $VAV2^{Onc}$-regulated RBF (top) or the RP gene (bottom). The Mantel-Cox test *P* value is indicated for each transcript.

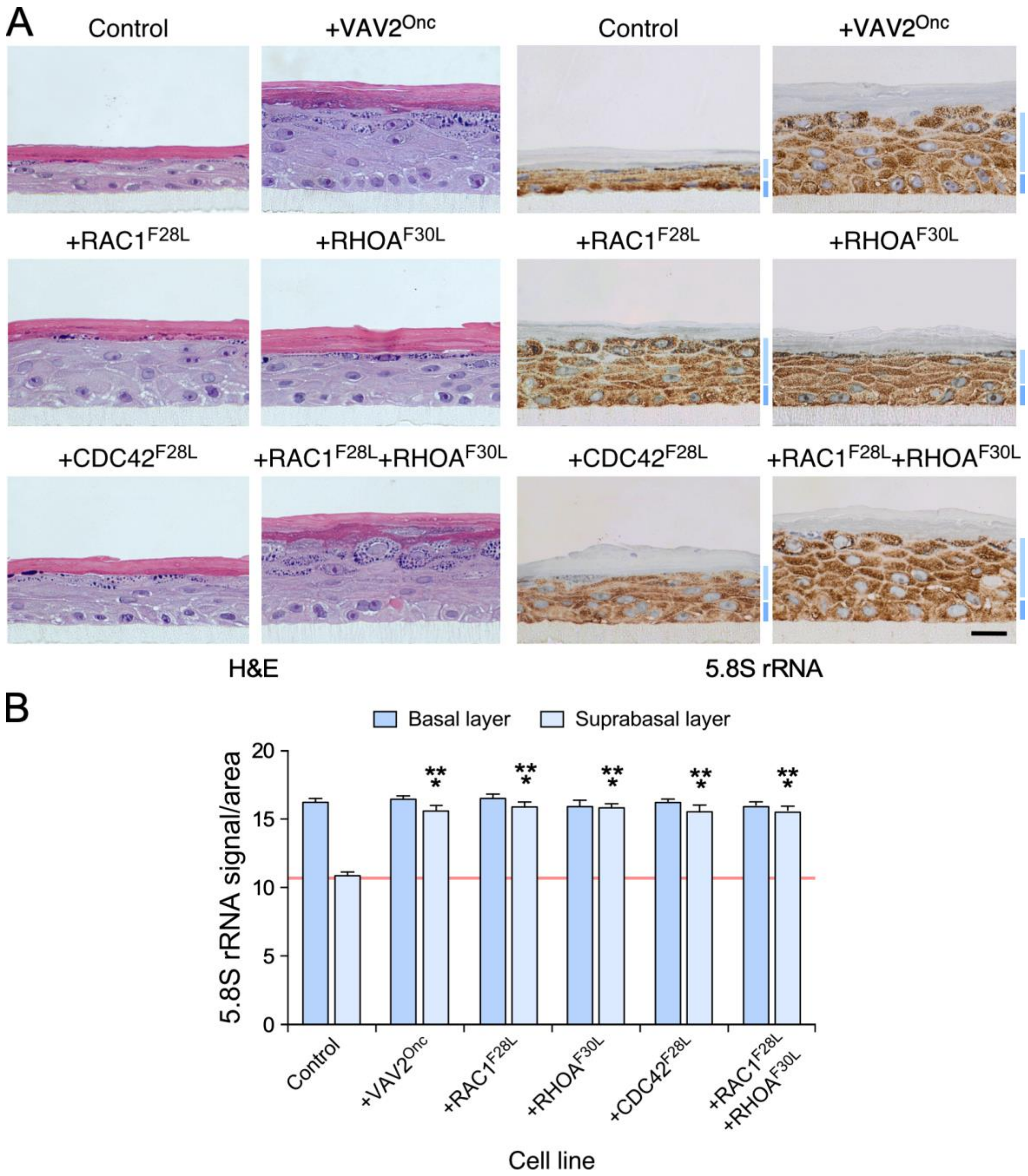

SUPPLEMENTARY FIGURE 2. Activated RHO proteins also stimulated ribosome biogenesis

(A) Representative images of organotypic cultures of human keratinocytes expressing the indicated proteins (top) that were stained with either hematoxylin-eosin (H&E) (two left columns) or labeled with an antibody to the 5.8S rRNA plus hematoxylin (two right panels). Dark and light blue bars indicate the 5.8S rRNA immunoreactivity values found in the basal and suprabasal epithelial layers, respectively. Scale bar, 10 µm.

(B) Quantitation of the 5.8S rRNA immunoreactivity obtained in panel (A). ***, $P < 0.0001$ of test samples versus their respective layer control (ANOVA and Tukey's HSD tests, $n = 3$ independent experiments). Data represent the mean ± SEM. Source data for this figure are provided as a Source Data file.

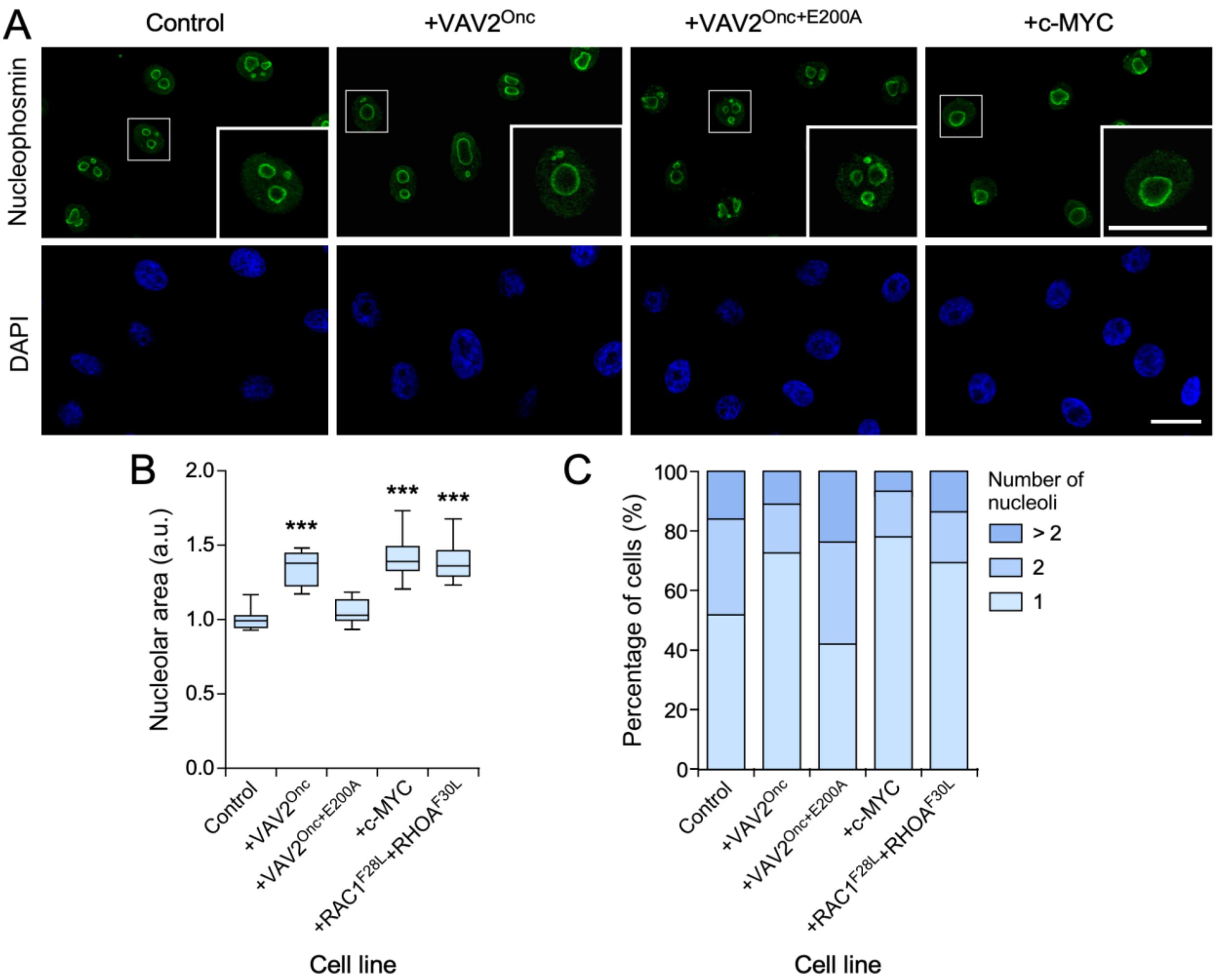

SUPPLEMENTARY FIGURE 3. Activation of the VAV2 pathway promotes changes in nucleolar morphology

(A) Representative confocal microscopy showing the type of nucleoli found in human keratinocytes expressing the indicated proteins (top). Nucleoli and nuclei were detected using immunostaining with the nucleolar protein nucleophosmin (top panels) and DAPI staining (lower panels), respectively. Scale bar, 20 µm. In the top panels, the squares indicate the areas that are shown enlarged in the right bottom corner of each image.

(B and C) Quantitation of the area (B) and number (C) of nucleoli found in keratinocyte samples analyzed in (A). ***, $P < 0.001$ (ANOVA and Dunnett's multiple comparison tests, $n = 3$ independent experiments). In the case of B, only cells with a single nucleolus were scored to allow comparisons in all conditions. Data represent the mean ± SEM. Source data for this figure are provided as a Source Data file.

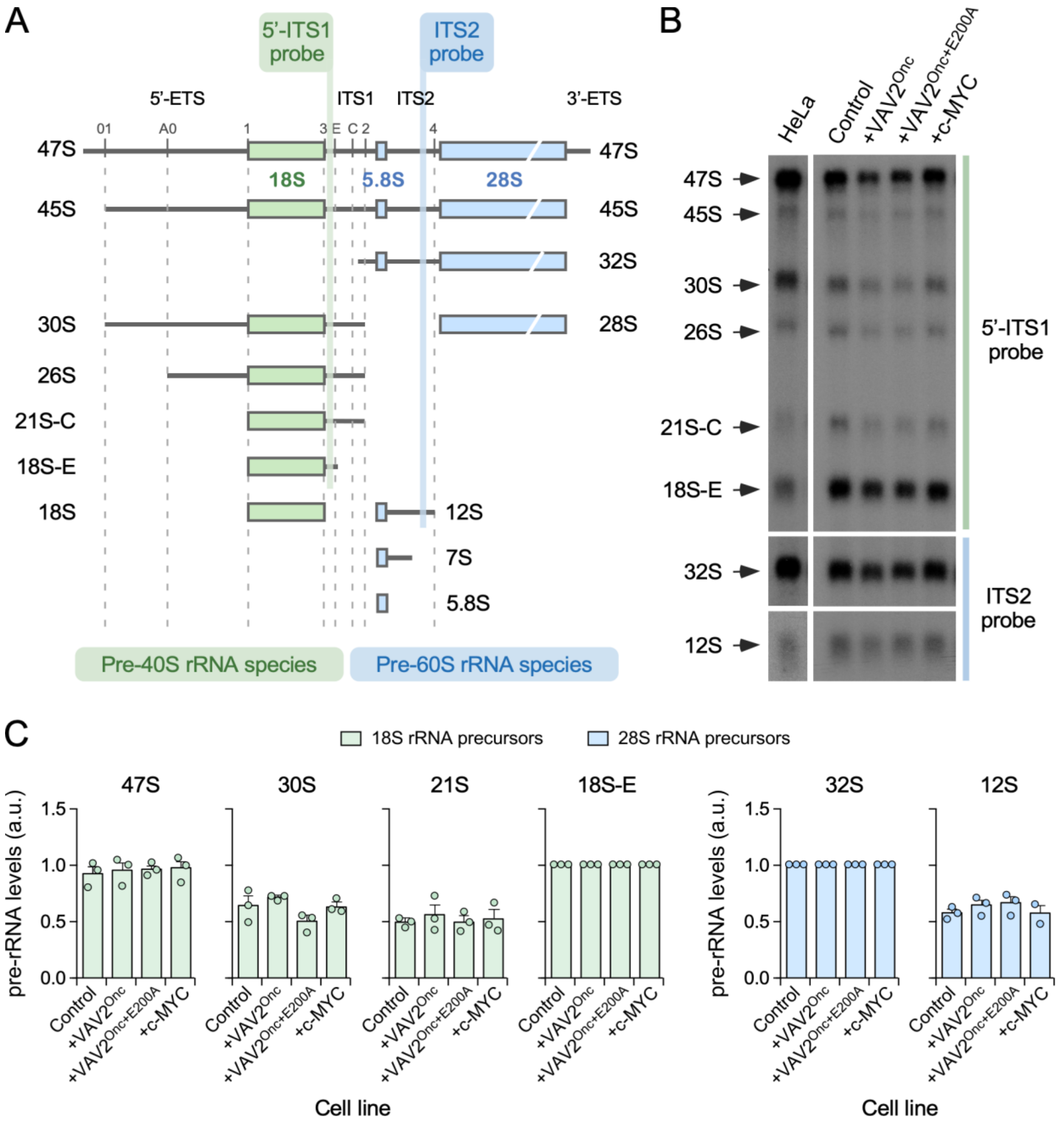

SUPPLEMENTARY FIGURE 4. VAV2^{Onc} does not induce changes in pre-rRNA processing in human keratinocytes

(A) Scheme depicting the pre-rRNA processing pathway in human cells. Pre-rRNA species belonging to the small (left, green) and large (right, blue) ribosome subunits can be identified by Northern blot using the 5′-ITS1 and the ITS2 probes (top), respectively. The size of the pre-rRNA species is indicated at the right and left of the figure. ETS, external pre-rRNA segment; ITS, internal pre-rRNA segment.

(B) Representative Northern blot analysis using total RNAs from HeLa cells (left column) and indicated human cells (rest of lanes) to detect the abundance of the pre-rRNA species that are recognized by the 5′-ITS and ITS2 probes (right). The sizes of the pre-rRNA species are indicated on the left.

(C) Quantitation of the relative abundance of the interrogated pre-RNA species in the keratinocyte cell lines analyzed in (B). Normalization was performed relative to the abundance of the 47S pre-RNA. Statistically nonsignificant differences were found between the indicated samples and controls (ANOVA and Dunnett's multiple comparison test, $n = 3$ independent experiments). Data represent the mean ± SEM. Source data for this figure are provided as a Source Data file.

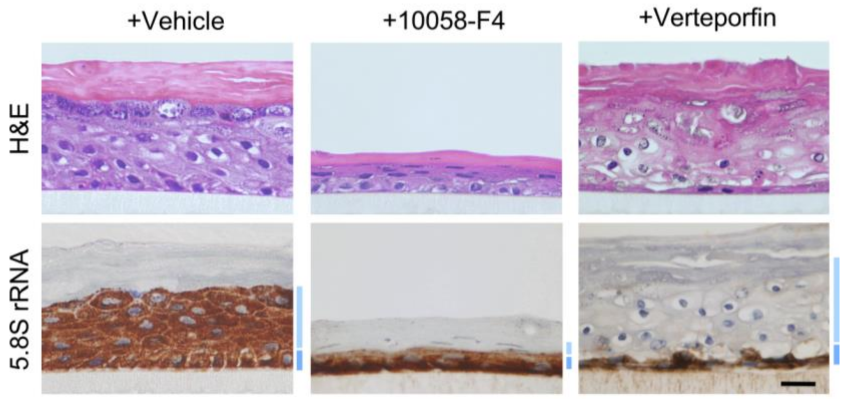

SUPPLEMENTARY FIGURE 5. Downstream VAV2 GTPases also promote increased synthesis of the pre-rRNA in a MYC- and YAP/TAZ-dependent manner

Representative confocal microscopy images of $RAC1^{F28L}$+$RHOA^{F30L}$-expressing human keratinocytes treated with the indicated inhibitors (top) that were stained with either hematoxylin-eosin (top panels) or with an antibody to the 5.8S rRNA plus hematoxylin (bottom panels). Dark and light blue bars indicate the basal and suprabasal epithelial layers, respectively (right). Scale bar, 10 µm. The quantitation of 5.8S rRNA immunoreactivity found in this and two additional experiments is shown in **Figure 3B** (right).

IMAGES OF THE ORIGINAL BLOTS SHOWN IN Fig. 2E

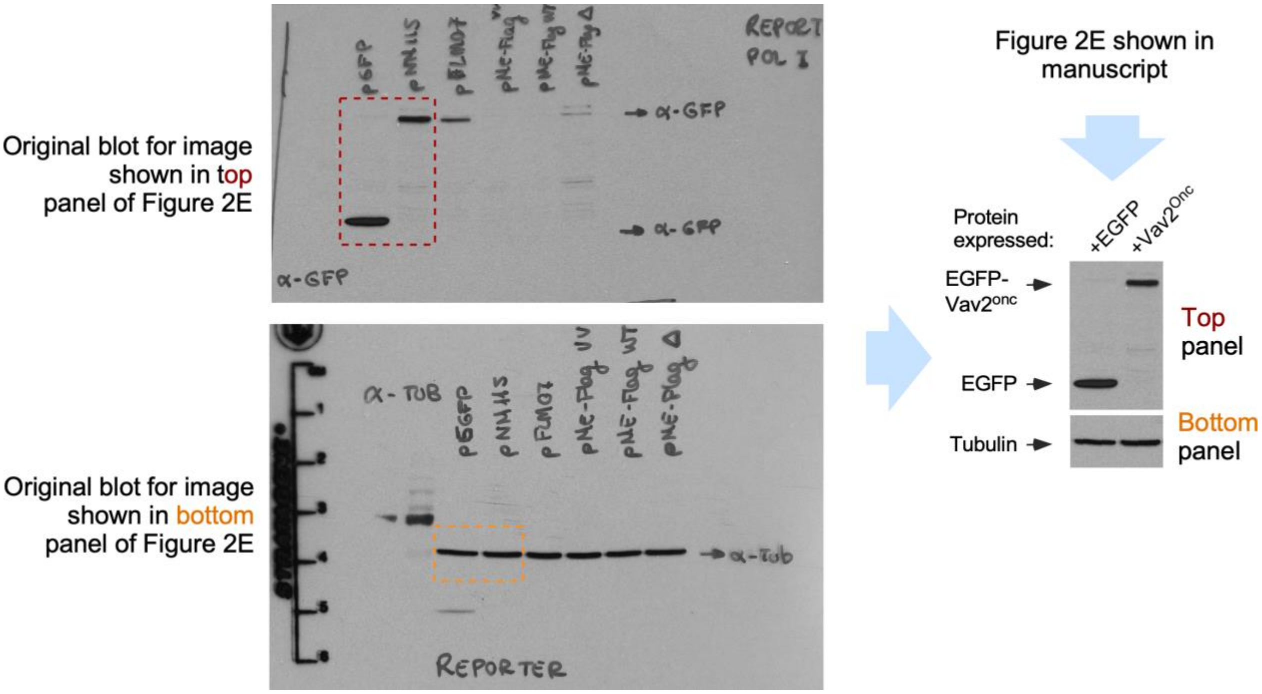

IMAGES OF THE ORIGINAL BLOTS SHOWN IN SUPPLEMENTARY Fig. 4B

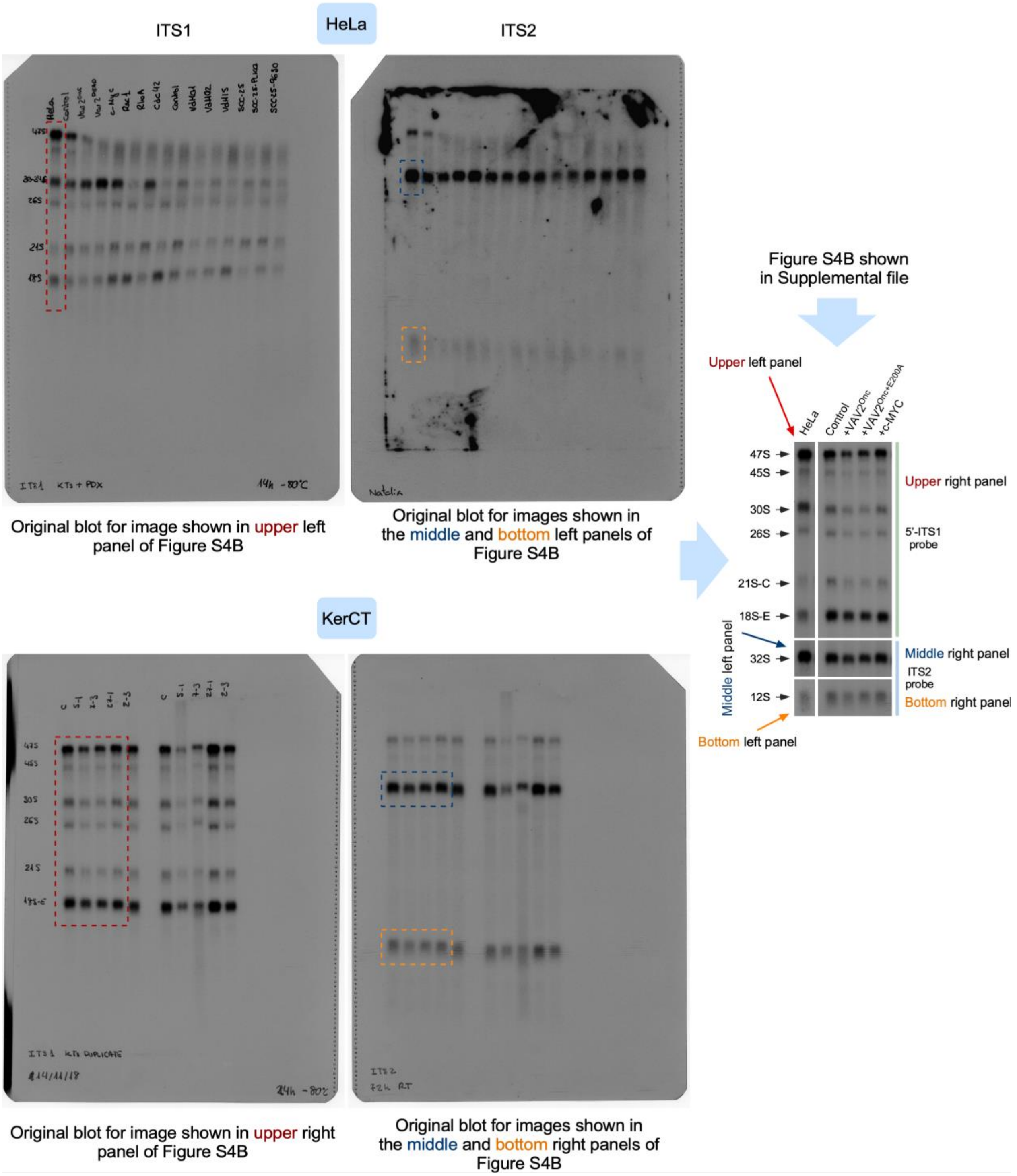

Supplement: Supplementary file 1 — Supplementary Figures. [file 41598_2024_54808_MOESM1_ESM.pdf]
